# Magnetically Retrievable Platinum Nanoreporters for Efficient Lateral Flow Immunoassay in Complex Bio‐Samples

**DOI:** 10.1002/smll.202506622

**Published:** 2025-11-28

**Authors:** Yuxi Cheng, Luca Panariello, Adam Creamer, Christy J. Sadler, André Shamsabadi, Kathleen Lupien, Ali Vaughan, Thomas Gervais, Molly M. Stevens

**Affiliations:** ^1^ Department of Materials Department of Bioengineering, and Institute of Biomedical Engineering Imperial College London London SW7 2AZ UK; ^2^ Department of Physiology, Anatomy and Genetics Department of Engineering Science, and Kavli Institute for Nanoscience Discovery University of Oxford Oxford OX1 3QU UK; ^3^ Department of Medical Biochemistry and Biophysics Karolinska Institutet Stockholm 171 11 Sweden; ^4^ Department of Engineering Physics Polytechnique Montréal Montréal H3T 1J4 Canada; ^5^ Nuffield Department of Medicine University of Oxford Oxford OX3 9DU UK; ^6^ NIHR Oxford Biomedical Research Centre University of Oxford Oxford OX3 9DU UK

**Keywords:** lateral flow immunoassay, magnetic separation, matrix effect, platinum nano‐catalysts

## Abstract

Lateral flow immunoassays (LFIAs) are widely used for point‐of‐care diagnostics, but their development is challenged by the complexity and variability of patient samples. In particular, LFIAs often exhibit reduced sensitivity and specificity when used with patient samples, compared to their performance with analyte‐spiked idealized matrices. Patient samples are inherently complex, with variations in physical and biochemical properties between patients. This complexity has consequences for the performance of LFIAs, and can result in non‐specific binding on the test line, discoloration of the nitrocellulose membrane, and incomplete sample flow along the test strip. To address these challenges, a magnetically retrievable platinum nanoreporter (termed Pt@Fe_3_O_4_) is developed for LFIAs. Leveraging the magnetic properties of the Fe_3_O_4_ core, magnetic separation is utilized to enable the purification and concentration of target antigens from complex human matrices, including serum, saliva, and even stool samples. This also eliminates assay inconsistencies caused by inter‐sample variability. Further, the suitability of Pt@Fe_3_O_4_ nanoreporters has been explored for use as detection probes in LFIAs. Signal enhancement is demonstrated by the utilization of the magnetic and enzyme‐mimicking activity of the nanoreporter, resulting in a marked improvement in sensitivity, as evidenced by a 2‐ to 4‐fold decrease in the visual limit of detection.

## Graphical abstract

Lateral flow immunoassays (LFIAs) for rapid, point‐of‐care testing face challenges with real patient samples. This work presents magnetically retrievable platinum nanoreporters for LFIAs that purify and concentrate samples, and amplify signals, enhancing sensitivity and lowering the visual limit of detection in complex bio‐samples such as human serum, saliva, and stools.

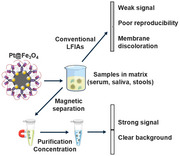

## 1 Introduction

Lateral flow immunoassays (LFIAs) are tests widely used for diagnostics at the point‐of‐care (POC) owing to their simplicity, rapid time to results, and cost‐effectiveness.^[^ ^1^, ^2^ ^]^ Nanoreporters in LFIAs typically consist of nanoparticles functionalized with affinity agents, such as antibodies, that bind specifically to the target. This enables visualization of the result through color changes^[^ ^3^, ^4^ ^]^ or other signals (e.g., fluorescence intensity)^[^ ^5^ ^]^ at the test line.

The clinical application of LFIAs involves employing patient samples, such as serum, saliva, or stool.^[^ ^6^, ^7^, ^8^ ^]^ Patient samples are complex matrices, where the biochemical composition can vary drastically between patients. These samples contain varying concentrations of cells, proteins, lipids, and other macromolecules that can interact with the nanoreporters of the LFIA device. These variations are influenced by numerous factors, including the time of sample collection, ethnicity, age, gender, lifestyle, pathophysiological state, and medication. In LFIAs, the presence of biological molecules can cause nanoreporters to aggregate, forming clusters that may obstruct or interfere with the normal flow and detection of the antigens.^[^ ^9^, ^10^ ^]^ Additionally, the biomolecular components in the sample matrix can lead to non‐specific interactions between the nanoreporters and the nitrocellulose membrane, as well as the capture affinity agent of the LFIA device. Such interactions contribute to a high background along the nitrocellulose membrane and non‐specific binding to the test line (false positives).^[^ ^11^ ^]^ The inherent heterogeneity between samples from different donors exacerbates the challenges posed by matrix effects in LFIAs, potentially compromising their accuracy.^[^ ^12^, ^13^, ^14^, ^15^, ^16^, ^17^, ^18^ ^]^ This is particularly crucial when developing LFIAs with quantitative readouts, such as for the detection of C‐reactive protein or specific cancer biomarkers (e.g., carcinoembryonic antigen or prostate‐specific antigen).^[^ ^19^, ^20^ ^]^

A further challenge faced by LFIAs is the highly variable physical properties of biological matrices, in particular, viscoelastic properties. Sample viscosity is highly relevant to LFIA applications, as it significantly affects the fluid dynamics of sample wicking through the test strip. Increased viscosity can slow the fluid flow along the strip, reducing the mass transfer of the nanoreporters to the test lines.^[^ ^21^ ^]^ Furthermore, high viscosity can lead to non‐homogeneous sample flow, causing uneven distribution and potential clogging of the assay.^[^ ^12^, ^22^, ^23^, ^24^ ^]^

Despite the significant impact of matrix effects in LFIAs, this issue is often overlooked in the earliest stages of LFIA development and is only addressed during translation. This leads to significant challenges in the effective deployment of new nanoreporters. Typically, patient samples are diluted with optimized running buffers to standardize viscosity and reduce the concentration of interfering substances.^[^ ^6^, ^25^, ^26^, ^27^, ^28^, ^29^ ^]^ However, the dilution of patient samples also reduces the effective concentration of target analytes used in the LFIA, therefore decreasing the sensitivity of the assay. Use of external filtration devices or addition of filtration membranes into the sample pad of the LFIA strip are other strategies used to mitigate matrix effects. However, these approaches may increase the complexity and cost of the device, and do not always achieve sufficient purification.^[^ ^30^ ^]^

To overcome low assay sensitivity, nanoreporters have been engineered to amplify the observable signal, lowering the limit of detection (LOD) and improving analytical sensitivity. One example is the use of magnetic nanoparticles, such as iron oxide nanoparticles. Due to their intrinsic magnetic properties, these nanoparticles can facilitate the isolation and concentration of target analytes by magnetic separation from a patient sample, thereby significantly improving the sensitivity of LFIAs.^[^ ^6^, ^8^, ^31^, ^32^, ^33^ ^]^ Less explored in the field are the advantages deriving from the use of magnetic separation to remove unwanted components and reduce sample‐to‐sample variability inherent in biological matrices. Further strategies have aimed at amplifying the signal generated at the test line through the use of catalytically active nanoreporters. This includes nanoparticles with enzyme‐mimicking activity, such as platinum nano‐catalysts (PtNCs), which exhibit peroxidase‐mimicking activity. Previous work has demonstrated the application of such nanoparticles in LFIAs, generating an improved signal at the test line through oxidation of chromogenic substrates, such as 4‐Chloro‐1‐naphthol/3,3′‐Diaminobenzidine (CN/DAB).^[^ ^34^, ^35^ ^]^

**Scheme 1:**
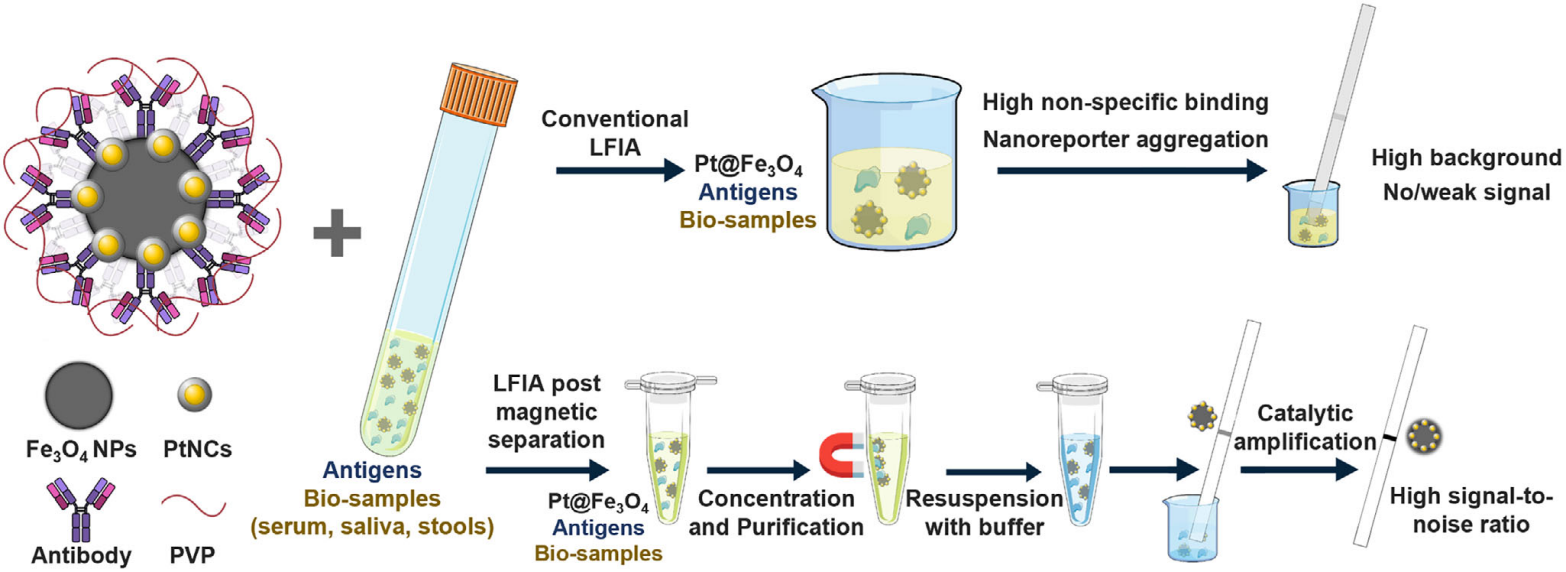
Illustration of LFIA for antigen detection in biological matrices without or with magnetic separation. Conventional LFIAs using unprocessed biological matrices often encounter high non‐specific binding and nanoreporter aggregation, leading to high background and even complete loss of signal. In comparison, LFIAs with Pt@Fe_3_O_4_ nanoreporters enable the magnetic purification and concentration of analytes and catalytic amplification of the readouts, significantly enhancing sensitivity and allowing for a high signal‐to‐noise ratio.

Here, we showcase the successful development and integration of magnetically retrievable platinum nanoreporters (Pt@Fe_3_O_4_) as detection probes in LFIAs. The developed Pt@Fe_3_O_4_ nanoreporters can address some of the shortcomings associated with the direct use of patient samples in LFIAs (**Scheme** **1**). Specifically, we highlight the use of Pt@Fe_3_O_4_ nanoreporters to purify and concentrate the target analytes from complex bio‐samples. After magnetic recovery, the enriched materials can be resuspended in optimized assay buffer prior to running the LFIA. The developed nanoreporters also enable further signal amplification through enzyme‐mimicking reactions. The use of magnetic separation enables us to perform LFIAs using very challenging matrices as saliva and stool, without the need for any dilution, while commercial products require sample dilution up to a factor of 20. Moreover, we show how the use of Pt@Fe_3_O_4_ nanoreporters effectively minimizes variability in assay results arising from inter‐sample heterogeneity, altering fluid flow during the assay, as supported by numerical simulations. The proposed Pt@Fe_3_O_4_ nanoreporter provides a promising platform for the design of future LFIA nanoreporters to reduce matrix effects and improve assay robustness and reproducibility.

## 2 Results and Discussion

### 2.1 Synthesis and Characterization of Pt@Fe_3_O_4_

**Figure 1:**
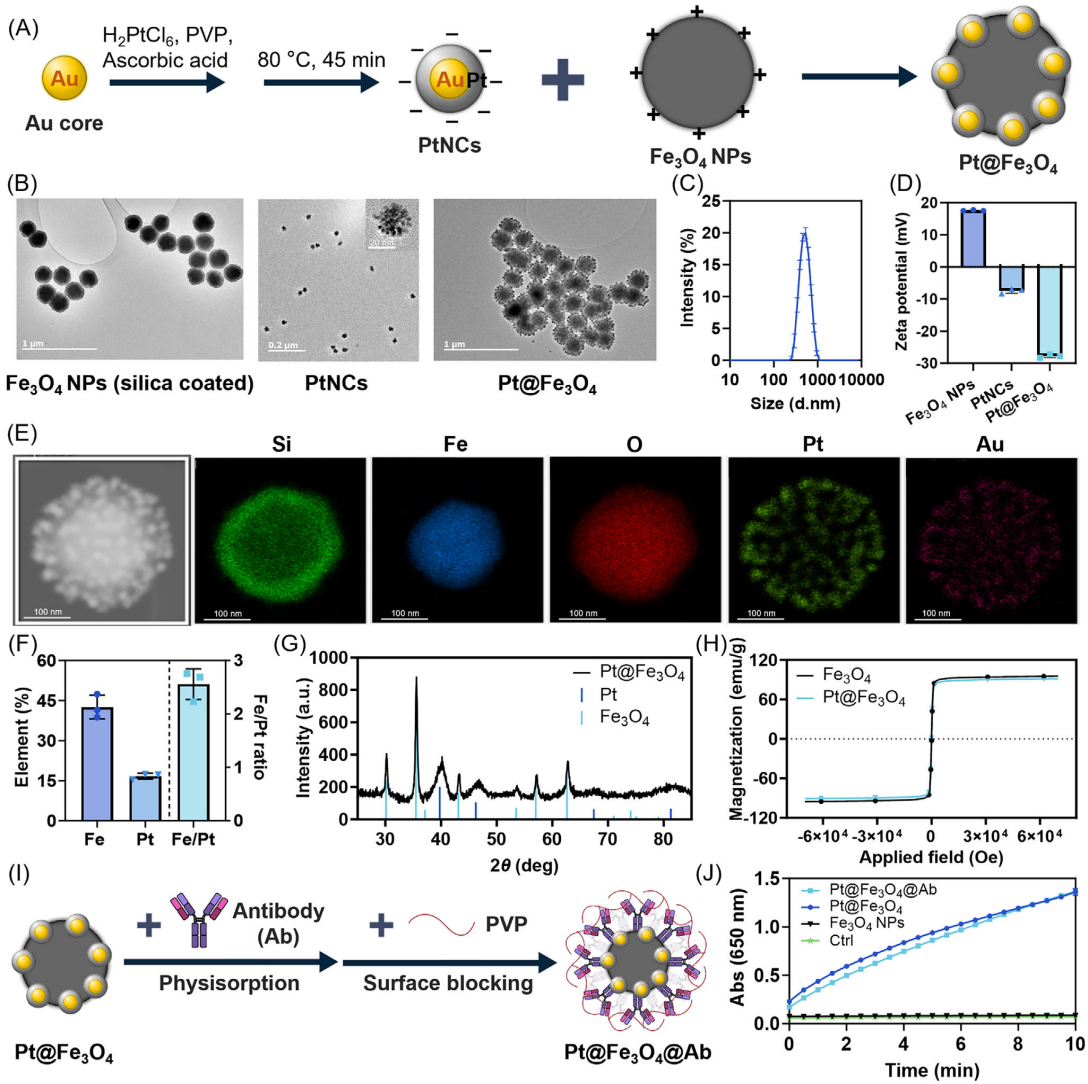
A) Schematic illustration showing the synthesis process for Pt@Fe_3_O_4_. B) TEM images of silica‐coated Fe_3_O_4_ NPs, PtNCs, and Pt@Fe_3_O_4_. Scale bars for Fe_3_O_4_ NPs and Pt@Fe_3_O_4_ are 1 µm, for PtNCs are 0.2 µm or 20 nm (for the zoomed‐in image). C) Hydrodynamic diameter distribution of Pt@Fe_3_O_4_ measured by dynamic light scattering (DLS). Data shown as mean ± S.D. (*n* = 3 measurements). D) Zeta potential of Fe_3_O_4_ NPs, PtNCs, and Pt@Fe_3_O_4_. Data shown as mean ± S.D. (*n* = 3 measurements). E) Scanning transmission electron microscopy (STEM)(imaging and EDS mapping showing the morphology of Pt@Fe_3_O_4_ and the distribution of Si, Fe, O, Pt, and Au among the complexes (scale bar, 100 nm). F) Elemental composition of Pt@Fe_3_O_4_ quantified by ICP‐MS. Data shown as mean ± S.D. (*n* = 3 independently synthesized batches of Pt@Fe_3_O_4_). G) Crystal structure of Pt@Fe_3_O_4_. The lines below the experimentally obtained XRD pattern show the peak positions of the reference patterns (PDF 01‐088‐0866 for Fe_3_O_4_ and PDF 00‐004‐0802 for Pt). H) Magnetization curves of Fe_3_O_4_ NPs and Pt@Fe_3_O_4_ normalized to the amount of iron present in each sample. I) Schematic illustration showing the antibody conjugation on the surface of Pt@Fe_3_O_4_. J) Time‐resolved evolution of the absorbance at 650 nm caused by the oxidation of TMB in the presence of H_2_O_2_ catalyzed by Pt@Fe_3_O_4_@Ab, Pt@Fe_3_O_4,_ and Fe_3_O_4_ NPs. DPBS was used as the control condition. Data shown as mean ± S.D., *n* = 3 separate plate wells, with a final nanoparticle concentration of 0.2 pm.

The Pt@Fe_3_O_4_ were synthesized by decorating a positively charged magnetic core with negatively charged PtNCs, as illustrated in **Figure** **1**A. Peroxidase‐mimicking nanozymes composed solely of Pt show poor stability, and most Pt atoms remain unexposed and catalytically inactive. Therefore, bimetallic core–shell nanospheres with a Pt shell, particularly those with a gold core, have been extensively studied, as gold may influence the catalytic activity of Pt.^[^ ^36^, ^37^ ^]^ Here, PtNCs with Au seeds were synthesized following a previously reported protocol with minor modifications.^[^ ^34^ ^]^ This method enables precise control of PtNCs size and monodispersity (hydrodynamic diameter ≈15 nm, Figure S1A, Supporting Information) by tuning the Au seed size and H_2_PtCl_6_ amount, while the non‐conformal growth of Pt on Au produces a porous dendritic structure that provides additional surface area and may enhance catalytic activity.^[^ ^34^, ^38^ ^]^ The energy dispersive X‐ray spectroscopy (EDS) mapping data in Figure S1B (Supporting Information) illustrate that these PtNCs consist of an Au core with a porous platinum outer layer. Commercially available silica coated Fe_3_O_4_ nanoparticles (Fe_3_O_4_ NPs) were employed as the magnetic component for Pt@Fe_3_O_4_, with a diameter of ≈300 nm (Figure S1C, Supporting Information), and EDS mapping demonstrates the distribution of Si, Fe and O, clearly indicating a core–shell structure, with Fe and O corresponding to the Fe_3_O_4_ core and Si and O to the silica shell (Figure S1D, Supporting Information). Pt@Fe_3_O_4_ were then synthesized by simply incubating Fe_3_O_4_ NPs with PtNCs on a shaker overnight. The morphology of the PtNCs, Fe_3_O_4_ NPs, and Pt@Fe_3_O_4_ complexes was investigated using transmission electron microscopy (TEM) (Figure 1B). The size of the newly formed complex between Fe_3_O_4_ NPs and PtNCs remained ≈300 nm (Figure 1C), suggesting that the PtNCs formed a monolayer on the surface of the Fe_3_O_4_ core. The binding between PtNCs and Fe_3_O_4_ NPs was primarily driven by electrostatic surface interactions, as the Fe_3_O_4_ NPs displayed a net positive surface charge, while the PtNCs displayed a negative zeta potential (Figure 1D). Following complex formation, the surface potential of the Pt@Fe_3_O_4_ became highly negative (Figure 1D), indicating a high‐density binding of PtNCs on the Fe_3_O_4_ core. EDS mapping of Pt@Fe_3_O_4_ (Figure 1E) shows the distribution of Si, Fe, O, Pt, and Au within the complex, confirming the core–shell structure of the Fe_3_O_4_ NPs and the surface‐bound PtNCs, with Au cores and Pt shells. Notably, the binding of PtNCs to Fe_3_O_4_ NPs enhanced the extinction cross section in the visible light range of the Pt@Fe_3_O_4_ compared to that of the pure Fe_3_O_4_ NPs (Figure S2A, Supporting Information).

The elemental composition of Pt@Fe_3_O_4_ was analyzed using inductively coupled plasma mass spectrometry (ICP‐MS), revealing that Pt@Fe_3_O_4_ consisted of ≈43% Fe and 17% Pt, as illustrated in Figure 1F. To assess the activity of Pt@Fe_3_O_4_, which determines the catalytic signal amplification capability of the nanoreporters, their peroxidase‐mimicking catalytic property was evaluated by monitoring the oxidation of 3,3′,5,5′‐tetramethylbenzidine (TMB) in the presence of H_2_O_2_, producing blue oxidized products with an absorbance peak at around 650 nm, as shown by the schematic representation in Figure S2B (Supporting Information).^[^ ^39^ ^]^ The ICP‐MS results, together with the particle size (Figure S2C, Supporting Information) and catalytic activity data (Figure S2D, Supporting Information) obtained from three independent particle batches, show almost no variation, demonstrating the good reproducibility of the synthesis. Further experimental data demonstrate that the Pt@Fe_3_O_4_ nanoreporter achieves specific activity (*SA*, 8436 U mg^−1^, Figure S2E, Supporting Information)^[^ ^40^ ^]^ and catalytic efficiency (*K_cat_*, 7.94 × 10^9^ s^−1^, Figure S2F, Supporting Information)^[^ ^41^ ^]^ that significantly outperform those reported for a wide range of comparable nanozymes. X‐ray diffraction (XRD) analysis in Figure 1G further indicated that Pt@Fe_3_O_4_ exhibited a combined crystal structure characteristic of both Fe_3_O_4_ NPs and PtNCs, with the clear presence of peaks characteristic of both Fe_3_O_4_ and Pt. The Pt peaks appear broader than those of Fe_3_O_4_, suggesting a larger crystallite size for the iron oxide component than for the Pt, consistent with the particle size of the different elements of the Pt@Fe_3_O_4_. The magnetization curves (Figure 1H) demonstrate that these nanoreporters exhibit a strong magnetic response, with a saturation magnetization around 90 emu g_Fe_^−1^. Both Fe_3_O_4_ NPs and Pt@Fe_3_O_4_ displayed similar saturation magnetization, indicating that the Pt shell did not significantly affect the magnetic properties of the new nanoreporters. Compared with freshly prepared Pt@Fe_3_O_4_, the nanoparticles showed no significant changes in stability (hydrodynamic size, Figure S2C, Supporting Information) or catalytic activity (Figure S2D, Supporting Information), even after long‐term storage, drying, or exposure to harsh biological conditions (extended incubation time and elevated temperature). These results highlight their strong potential for clinical translation and practical usability.

### 2.2 Antibody Conjugation on Pt@Fe_3_O_4_ and Characterizations

The Pt@Fe_3_O_4_ was then implemented in a simplified half dipstick lateral flow sandwich immunoassay, in which detection antibodies were attached to the surface of Pt@Fe_3_O_4_, while capture antibodies were printed onto a nitrocellulose membrane, attached beneath an absorbent pad.^[^ ^42^ ^]^ The conjugation of antibodies to Pt@Fe_3_O_4_ (Pt@Fe_3_O_4_@Ab) was achieved through physisorption, followed by surface blocking with polyvinylpyrrolidone (PVP) to minimize potential non‐specific interactions, as illustrated in Figure 1I. This antibody conjugation had minimal impact on the hydrodynamic size and zeta potential of the Pt@Fe_3_O_4_ nanoreporter (Figure S2G,H, Supporting Information, respectively). Previous studies reported that PtNCs (120 nm in diameter) used as LFIA nanoreporters lost 65% of their catalytic activity after antibody conjugation through direct electrostatic adsorption on the particle surface.^[^ ^34^ ^]^ Interestingly, the conjugated antibody layer did not affect the catalytic activity of Pt@Fe_3_O_4_ (Figure 1J). This suggests that the antibody adsorbed on the iron oxide surface is possibly due to its local positive charge, rather than individual PtNCs, thus presenting minimal restrictions on substrate access and other potential interferences.

### 2.3 Application of Pt@Fe_3_O_4_ for Antigen Detection in Human Serum

After the successful synthesis of the Pt@Fe_3_O_4_ nanoreporters and antibody conjugation, we sought to test them in LFIAs, challenging the reporters with several complex bio‐samples. First, we tested the magnetic separation efficiency (i.e., fraction of recoverable particles). The separation protocol proved to be robust and reproducible, achieving a recovery rate of ≈65% in human serum and saliva within 20 min, as shown in the quantitative data of recovered Pt in Figure S2I (Supporting Information). The fraction can likely be increased by increasing separation time; however, we fixed this timescale as a timely result is a key attribute for POC devices following the REASSURED criteria.^[^ ^43^, ^44^ ^]^ Notably, the peroxidase‐mimicking activity of Pt@Fe_3_O_4_ remained unaffected by biological components after magnetic separation from the matrices and redispersion in running buffer. This is demonstrated by the curves in Figure S2J (Supporting Information), which show identical kinetics of TMB oxidation catalyzed by Pt@Fe_3_O_4_ separated from different matrices. This confirms the effectiveness of the magnetic separation procedure in preserving the signal amplification activity of Pt@Fe_3_O_4_ nanoreporters in biological samples.

**Figure 2:**
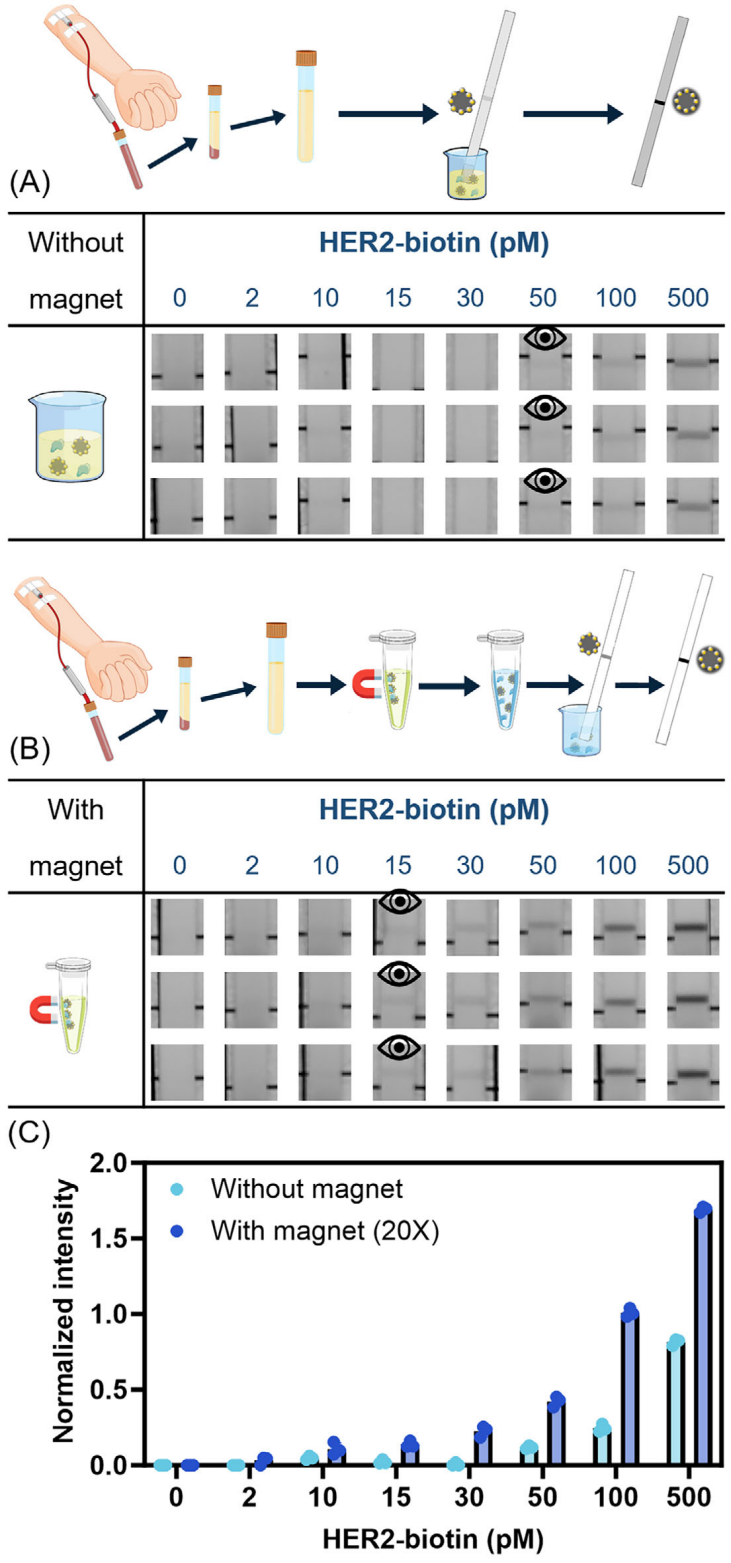
A) Detection of HER2‐biotin with Pt@Fe_3_O_4_@Ab in human serum without magnetic separation. The eye icons represent the visual LOD. B) Detection of HER2‐biotin with Pt@Fe_3_O_4_@Ab in human serum with magnetic separation and 20‐fold volumetric concentration (20×) of the sample. For each magnetic separation procedure, the particles were separated from 1400 µL of human serum and resuspended in 70 µL of running buffer. The eye icons represent the visual LOD. C) Normalized intensity of the lateral flow strip test lines in the detection of HER2‐biotin with Pt@Fe_3_O_4_@Ab in human serum without or with magnetic separation and 20‐fold volumetric concentration of the sample. Data shown as mean ± S.D., *n* = 3 independent experiments (magnetic separation procedures) with different batches of nanoparticles. The concentrations shown in the figure represent the initial values, i.e., before magnetic separation and concentration.

Human serum, a common sample in clinical applications, was chosen as the initial model to evaluate the efficiency of Pt@Fe_3_O_4_ nanoreporters in LFIAs. Serum is often considered a challenging matrix in LFIAs, as particle instability and non‐specific binding are often observed when transferring nanoreporters from optimized running buffers.^[^ ^6^, ^33^, ^45^, ^46^, ^47^ ^]^ HER2 (human epidermal growth factor receptor 2), a marker protein overexpressed in a subset of breast and ovarian cancers, was selected as a model antigen for detection in human serum.^[^ ^48^ ^]^ To streamline the experimental design, a HER2‐biotin conjugate was used to allow a printed polystreptavidin line to function as a capture antibody mimic. Trastuzumab, a HER2 antibody, was functionalized on the surface of Pt@Fe_3_O_4_. As shown in **Figure** **2**, Pt@Fe_3_O_4_ performed effectively in detecting HER2‐biotin in human serum both with and without the magnetic separation step. Further, applying the magnetic separation procedure and concentrating the analytes 20‐fold significantly enhanced the detection sensitivity, improving the visual LOD by more than threefold. The assay showed similar performance in an optimized running buffer, without or with magnetic separation and subsequent 20‐fold volumetric concentration of the sample (Figure S3, Supporting Information), demonstrating the stability of Pt@Fe_3_O_4_ in serum.

### 2.4 Application of Pt@Fe_3_O_4_ for SARS‐CoV‐2 N‐Protein Detection in Artificial Saliva

After demonstrating the use of Pt@Fe_3_O_4_ in serum, we moved to investigate its use in saliva. While saliva appears as an attractive sample type for LFIAs, due to its non‐invasive and easily accessible nature,^[^ ^25^, ^49^ ^]^ its highly variable physical, chemical, and biological properties—such as pH, viscosity, and digestion enzyme contents—pose significant challenges. In particular, saliva viscosity fluctuates considerably between donors and over time, further complicating LFIA design.^[^ ^13^, ^14^, ^23^ ^]^ These variations, influenced by factors such as collection time, diet, ethnicity, age, gender, lifestyle, pathophysiological state, and medication intake, can impact the reliability and consistency of LFIA‐based diagnostics.^[^ ^50^, ^51^, ^52^ ^]^ Given its relevance in saliva‐based diagnostics, the SARS‐CoV‐2 nucleocapsid protein (N‐protein) was chosen as the model antigen for this part of the study. First, the efficiency of the Pt@Fe_3_O_4_‐LFIA platform was tested in detecting N‐protein using the running buffer as a matrix. In the magnetic separation assay, two strategies were employed to enhance the signal‐to‐noise ratio. The first involved increasing the concentration factor during separation from one to five, while the second utilized catalytic amplification. To this last purpose, the peroxidase substrates CN/DAB were employed, which, in the presence of H_2_O_2_, were oxidized by PtNCs, leading to the formation of dark precipitates on the test line and thus amplifying the colorimetric readout, as shown in the schematic representations of the reactions in Figure S4A,B (Supporting Information).^[^ ^34^ ^]^ Figures S5–S7 (Supporting Information) demonstrate that the Pt@Fe_3_O_4_ nanoreporters for N‐protein detection performed efficiently both without and with magnetic separation in running buffer. In the magnetic separation experiment, the signal was successfully enhanced either by increasing the analyte concentration or by employing catalytic amplification, without introducing additional background noise. To evaluate the specificity of our LFIA, we tested the cross‐reactivity of the selected antibody pair with three other influenza N‐proteins. As shown in Figure S8 (Supporting Information), at a concentration of 20 nm, only our target antigen (40588‐V08B) produced a distinct signal on the test line, while all other antigens yielded no false‐positive results.

**Figure 3:**
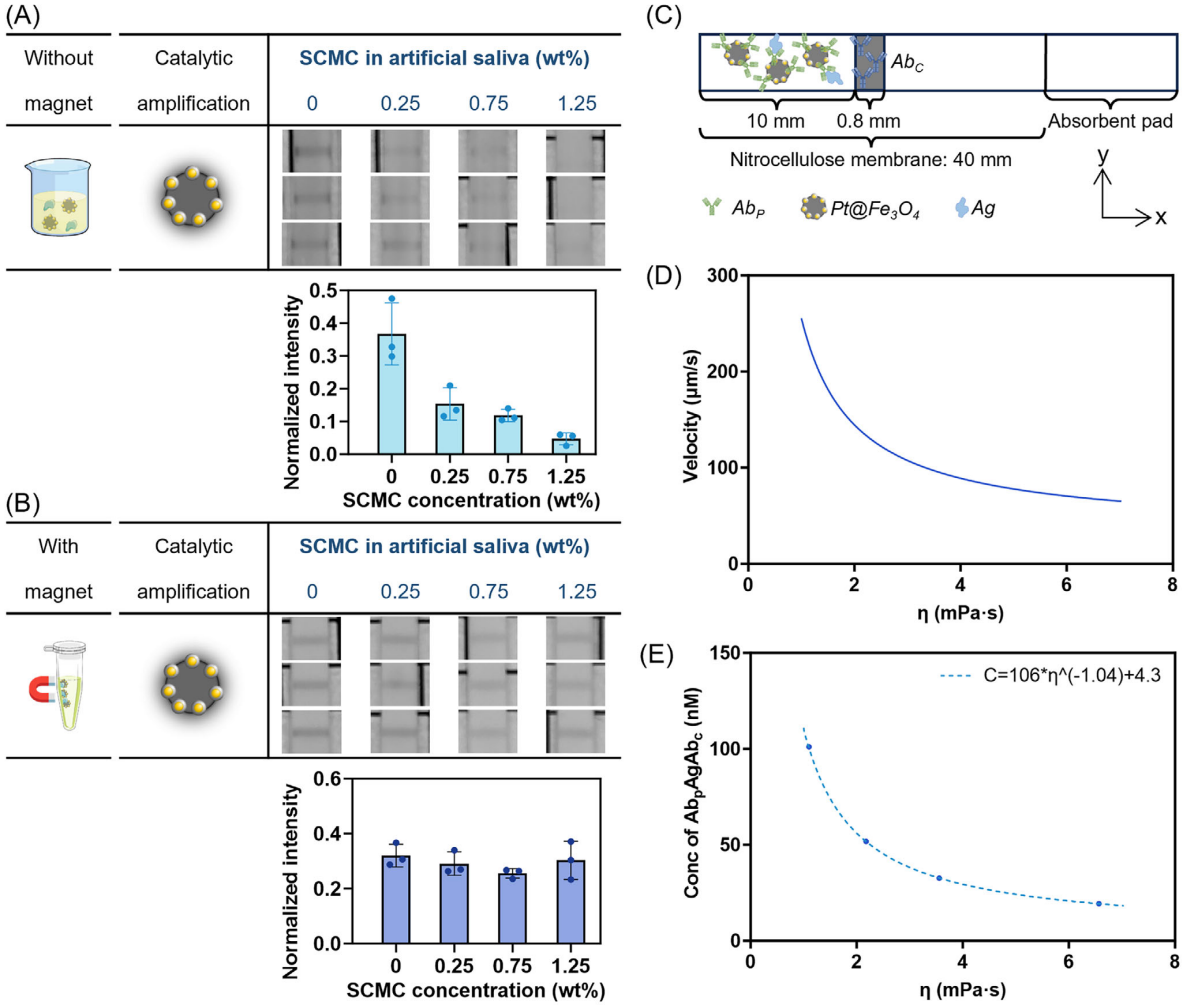
A) Detection of SARS‐CoV‐2 N‐protein with Pt@Fe_3_O_4_@Ab in artificial saliva without magnetic separation. Data shown as mean ± S.D., *n* = 3 independent experiments with different batches of nanoparticles. The LFIA signals obtained from artificial saliva with varying viscosities showed significant differences (Welch's ANOVA test, *p* = 0.0134). SCMC = sodium carboxymethyl cellulose. B) Detection of SARS‐CoV‐2 N‐protein with Pt@Fe_3_O_4_@Ab in artificial saliva with magnetic separation but no subsequent volumetric concentration. Data shown as mean ± S.D., *n* = 3 independent magnetic separation procedures with different batches of nanoparticles. For each magnetic separation procedure, the particles were separated from 200 µL of artificial saliva and resuspended in the same amount of running buffer. The LFIA signals obtained from artificial saliva with magnetic separation showed no significant difference among the groups (Welch's ANOVA test, *p* = 0.2612). The assays in A and B were conducted with 20 nm N‐protein and with CN/DAB amplification. C) Schematic illustration of the 2D model of Pt@Fe_3_O_4_ nanoreporter transport and reaction kinetics on an LFIA strip. D) Average convection velocity of Pt@Fe_3_O_4_ nanoreporter transport through the LFIA strip as a function of sample matrix viscosity. E) COMSOL simulated concentration of antigen delivered by Pt@Fe_3_O_4_ nanoreporters and captured on the test line after the full sample running period (10 min), as a function of sample matrix viscosity.

Due to the challenges posed by saliva's highly variable viscosity in LFIA applications,^[^ ^13^, ^14^, ^23^ ^]^ we investigated how viscosity affects the LFIA performance. Here, we mimicked saliva by preparing solutions of DPBS and different concentrations of sodium carboxymethyl cellulose (SCMC) to achieve varying viscosities. Rheometry analysis confirmed that the viscosity of artificial saliva increased with higher SCMC concentrations, matching the range expected for saliva,^[^ ^53^, ^54^ ^]^ as shown in the viscosity values η and η_∞_ in Figure S9A,B (Supporting Information). We then spiked N‐protein (20 nm) in and used Pt@Fe_3_O_4_ nanoreporters as a detection probe. The results in **Figure** **3**A reveal that the direct LFIAs (i.e., assay performed without magnetic separation, by simply having the sample wick through the membrane) were significantly impacted by the varying viscosity of the matrix, with lower signals observed when the viscosity increased. Statistical analysis further confirms that the LFIA signals obtained from artificial saliva samples with varying viscosities exhibited significant differences (Welch's ANOVA test, *p* = 0.0134). In contrast, when the magnetic separation procedure was applied, the signals across all viscosity groups became equivalent, as demonstrated in Figure 3B. This resulted in no statistically significant variation in LFIA signals among the groups (Welch's ANOVA test, *p* = 0.2612). Additionally, the recovery ratio and reproducibility of Pt@Fe_3_O_4_ nanoreporters through magnetic separation were not affected by the viscosity of the matrices (Figure S9C, Supporting Information). This result demonstrates that the Pt@Fe_3_O_4_ nanoreporters, combined with the magnetic separation procedure, can eliminate the influence of varying matrix viscosity on LFIAs.

To better understand how sample matrix viscosity influences the transport and reaction kinetics of Pt@Fe_3_O_4_ nanoreporters in LFIA, we developed a simplified 2D model (Figure 3C), assuming homogeneous flow along the z‐direction. Details of the parameters, variables, and values used in the simulations are listed in Table S1 (Supporting Information).

The LFIA process is simplified into two sequential reactions:

1. Antigen (*Ag*) binds to detection antibody (*Ab_p_*) on the nanoreporter to form a complex (*Ab_p_Ag*):
(1) $$Ab_{p}+Ag\to Ab_{p}Ag$$
2. The *Ab_p_Ag* complex is captured by immobilized capture antibodies (*Ab_c_*) at the test line:
(2) $$Ab_{p}\mathrm{Ag}+Ab_{c}\underset{k_{\mathrm{of}f_{c}}}{\overset{k_{on_{c}}}{↔}}Ab_{p}\mathrm{AgA}b_{c}$$

Equation (1) occurs in solution before entering the strip and is assumed to be at equilibrium, given the $k_{off_{p}}$ of 1.79 × 10^−5^ s^−1^, yielding an *Ab_p_Ag* complex half‐life of ≈11 h. Thus, only Equation (2) is considered during the test, assuming negligible *A* *g* desorption within the duration of the assay (*t_tot_* = 10 min). The concentration of *Ab_p_Ag* entering the strip is calculated using the equilibrium equation:

(3) $$k_{d_{p}}=\frac{\left({\left[Ab_{p}\right]}_{0}-\left[Ab_{p}Ag\right])({\left[Ag\right]}_{0}-\left[Ab_{p}Ag\right]\right)}{\left[Ab_{p}Ag\right]}$$

where [*Ab_p_*]_0_ is estimated from nanoparticle concentration *C_NP,_* and antibody equivalence (500 per nanoparticle). This yields [*Ab_p_Ag*] ≈ 0.68 nm, indicating that nearly all detection antibodies are saturated.

To determine the dominant transport mechanism, we compared characteristic times for nanoreporter diffusion, reaction, convection, and sedimentation across matrices of varying viscosities (Equations S1–S6, Supporting Information). The average diffusion time for a nanoparticle to reach an antibody was also estimated to ensure that particles diffuse quickly enough to be captured at the test line (Equation S7, Supporting Information).

Among all transport mechanisms, convection is identified as the dominant mode of nanoreporter flow through the strip, while diffusion and sedimentation are negligible.^[^ ^55^ ^]^ All characteristic time scales are summarized in **Table** **1**. Notably, convection velocity (Equation S5, Supporting Information) is found to be inversely related to viscosity, as shown in Figure 3D.

**Table 1:** Characteristic times for nanoreporter diffusion, reaction, convection, and sedimentation in the 2D LFIA model.

	Variable	η = 1.1 *mPa* · *s* [min]	η = 6.6 *mPa* · *s* [max]
Diffusion coefficient	*D_NP_*	1.30 × 10^−12^ >m^2^/s	2.17 × 10^−13^ m^2^/s
Diffusion time	*t_d_*	6 days	34 days
Reaction time	*t_r_*	0.205 s	0.205 s
Convection time	*t_c_*	3.40 s	11.94 s
Sedimentation time	*t_s_*	4878 s	29 630 s

At the test line, convection remains the limiting process as long as the available capture antibodies are not saturated. We assessed whether saturation occurs by calculating the total number of nanoparticles reaching the nitrocellulose membrane during the test via convection:

(4) $$N_{NP}=t_{tot}\cdot \bar{v_{c}}\cdot A\cdot \left[Ab_{p}Ag\right]=A\left(\frac{D_{w}t_{tot}}{2\eta L}+\frac{L}{2}\right)\left[Ab_{p}Ag\right]$$

where *A* = *h* · *w* is the cross‐sectional area of the strip.

The total number of capture antibodies on the test line is given by:

(5) $$N_{Ab_{c}}=C_{Ab_{c}}\cdot V=C_{Ab_{c}}\cdot h\cdot w\cdot e$$

Thus, the fraction of capture antibodies bound to *Ab_p_Ag* complexes is:

(6) $$\varepsilon =\frac{N_{NP}}{N_{Ab_{c}}}\propto \frac{1}{\eta }$$

For the fastest convective transport and highest capture fractions, which occur with the least viscous sample (η =  1.1 mPa · s), we calculate ɛ ≈ 2.98%, given *t_tot_* = 10 min. Since the majority of the capture antibody sites remain unoccupied (over 97%), the effective reaction time *t_r_* does not change significantly toward the end of the assay and remains much shorter than the convection time *t_c_*, confirming that the reaction is convection‐limited throughout the process.

As evident from Equation (4) and Figure 3D, *ɛ* is inversely proportional to the sample matrix viscosity (ɛ ∝ η^−1^). Given that the colorimetric signal is proportional to the amount of captured *Ab_p_Ag* complexes, the measured signal is also inversely proportional to viscosity, which is consistent with the trend observed in Figure 3A.

We further confirmed the proposed scaling with a numerical simulation performed using COMSOL. We implemented a 2D model with creeping flow and transport of diluted species modules. As shown in Figure 3E, the simulated Ag concentration captured on the test line after 10 min is indeed inversely correlated with sample viscosity, consistent with the theoretical predictions. These results indicate that variations in matrix viscosity significantly affect the reproducibility and reliability of LFIA results, underscoring the importance of our Pt@Fe_3_O_4_ nanoreporter combined with magnetic separation for use in complex bio‐samples, particularly in eliminating the influence of viscosity variability.

### 2.5 Application of Pt@Fe_3_O_4_ for SARS‐CoV‐2 N‐Protein Detection in Human Saliva

**Figure 4:**
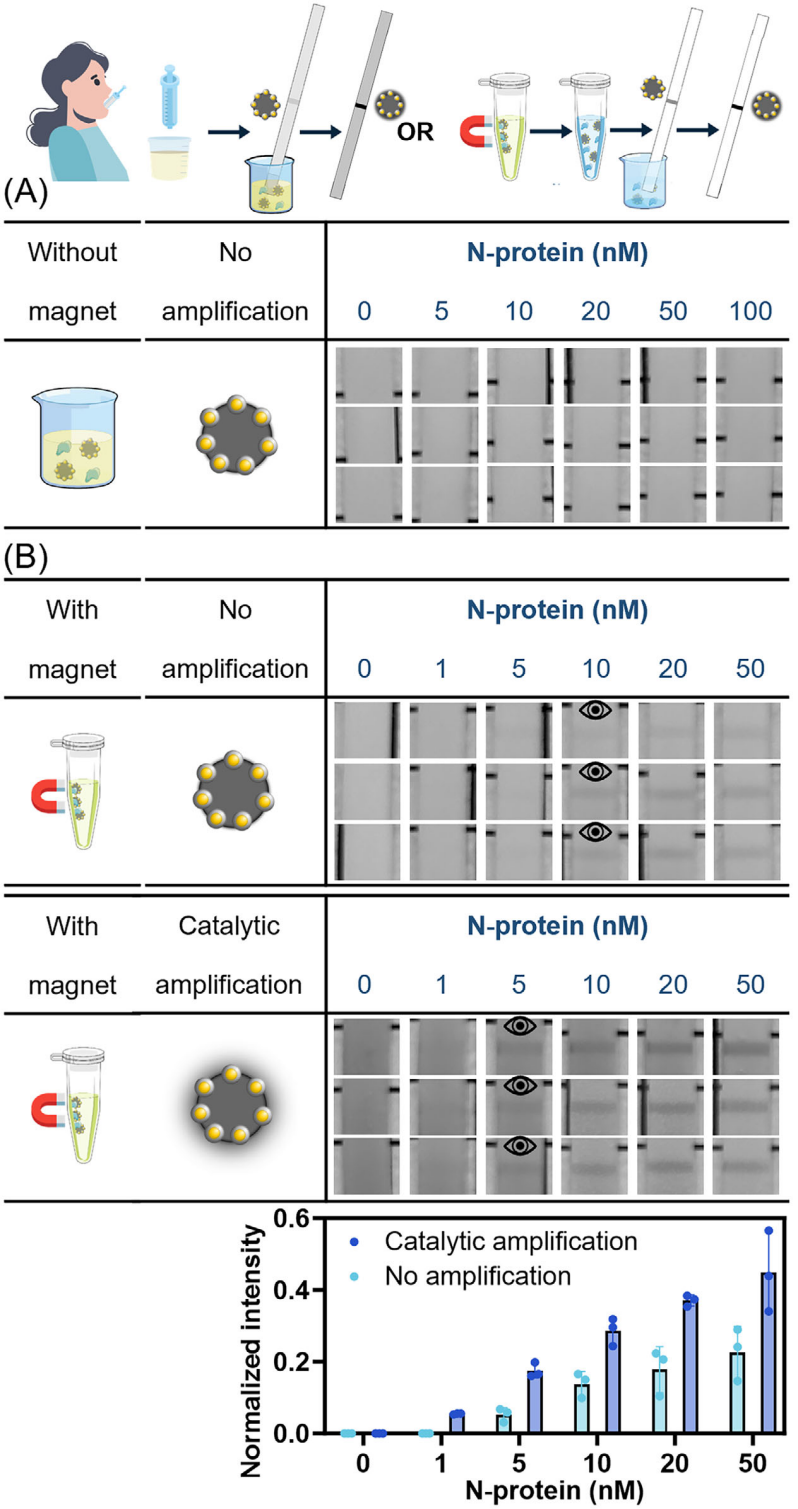
A) Detection of SARS‐CoV‐2 N‐protein with Pt@Fe_3_O_4_@Ab in human saliva without magnetic separation and without catalytic amplification. Data from three independent experiments with different batches of nanoparticles (*n* = 3). B) Detection of SARS‐CoV‐2 N‐protein with Pt@Fe_3_O_4_@Ab in human saliva with magnetic separation but no subsequent volumetric concentration, with or without catalytic amplification with CN/DAB substrates. Data shown as mean ± S.D., *n* = 3 independent magnetic separation procedures with different batches of nanoparticles. For each magnetic separation procedure, the particles were separated from 200 µL of human saliva and resuspended in the same amount of running buffer. The eye icons represent the visual LOD in these experiments.

We then applied our nanoreporters to human saliva. Surprisingly, using the direct LFIAs approach, no signal was observed, regardless of the N‐protein concentration or whether catalytic amplification was applied (see **Figure** **4**A for non‐amplified assay strips and Figure S10 (Supporting Information) for catalytically amplified assay strips). We hypothesized that magnetic separation could be used to mitigate this issue. As shown in Figure 4B, with the incorporation of the magnetic separation procedure after antigen capture in saliva, the test lines reappeared, indicating that the Pt@Fe_3_O_4_ nanoreporters successfully detected the N‐protein in human saliva, and the signals were further enhanced by catalytic amplification. This stark contrast highlights the critical role of magnetic separation in overcoming the matrix effect of saliva. This allows the Pt@Fe_3_O_4_ nanoreporters to flow through the LFIAs strips and form a sandwich structure with the N‐protein antigen and capture antibody on the test line for a colorimetric readout. This finding is particularly significant, as it demonstrates the necessity and effectiveness of combining Pt@Fe_3_O_4_ nanoreporters with magnetic separation to achieve successful antigen detection in LFIAs within complex biological matrices. Currently, only a few studies have investigated the use of magnetic or non‐magnetic nanoreporters for LFIA systems in saliva samples. However, most of them require saliva dilution with an optimized buffer.^[^ ^22^, ^25^, ^56^, ^57^, ^58^ ^]^ In this study, we demonstrate that, when combined with a magnetic separation procedure, the Pt@Fe_3_O_4_ nanoreporters could effectively capture antigens directly from undiluted saliva and produce a clear test line on a lateral flow strip. This, in turn, can lead to improved performance of the assay, as the dilution step would lead to a significant reduction in the effective concentration of antigens in the solution during the first capture step of the sandwich assay, one of the performance‐determining steps. Previous studies have also reported the use of magnetic nanoreporters for LFIA to detect antigens in undiluted saliva; however, such approaches typically require additional readout devices (e.g., UV lamps or portable fluorescence readers) rather than direct visual inspection, whereas our platform enables simple naked‐eye readout, which is particularly advantageous in resource‐limited settings.^[^ ^59^ ^]^

### 2.6 Application of Pt@Fe_3_O_4_ for Cholera Toxin Detection in Stool Samples

Encouraged by the results obtained in saliva, we decided to challenge our nanoreporters with stool samples. Human stool samples exhibit significant variability, not only between different individuals but also within the same individual over time, influenced by factors such as diet, water intake, gut microbiota composition, overall health status, and lifestyle choices.^[^ ^15^ ^]^ This variability includes the ratio of solid to liquid components and the concentration of various constituents, like undigested food residues, inorganic salts, pigments, fats and cholesterol, dead cells, and bacteria, making stool a highly complex matrix with complicated fluid properties. In this case, we selected cholera as a disease model: this is an acute diarrheal illness caused by *Vibrio cholerae*, transmitted through contaminated water or food. It leads to severe dehydration and can be fatal without prompt treatment. Rapid POC diagnostics are crucial for the timely identification and management of cholera outbreaks.^[^ ^60^, ^61^ ^]^ As one of the commonly used diagnostic methods in POC testing, currently available LFIA kits for cholera rapid diagnostic tests, such as the Crystal VC kit and the Bioline™ Cholera Ag O1/O139 test, require extensive dilution of stool samples to mitigate sample complexity,^[^ ^62^, ^63^ ^]^ which in turn significantly reduces the LOD. To simplify the experimental design and focus on investigating the performance of the nanoreporters through the LFIA strip, we used biotinylated cholera toxin beta subunit (CTB‐biotin) as a model antigen in the stool matrix.

**Figure 5:**
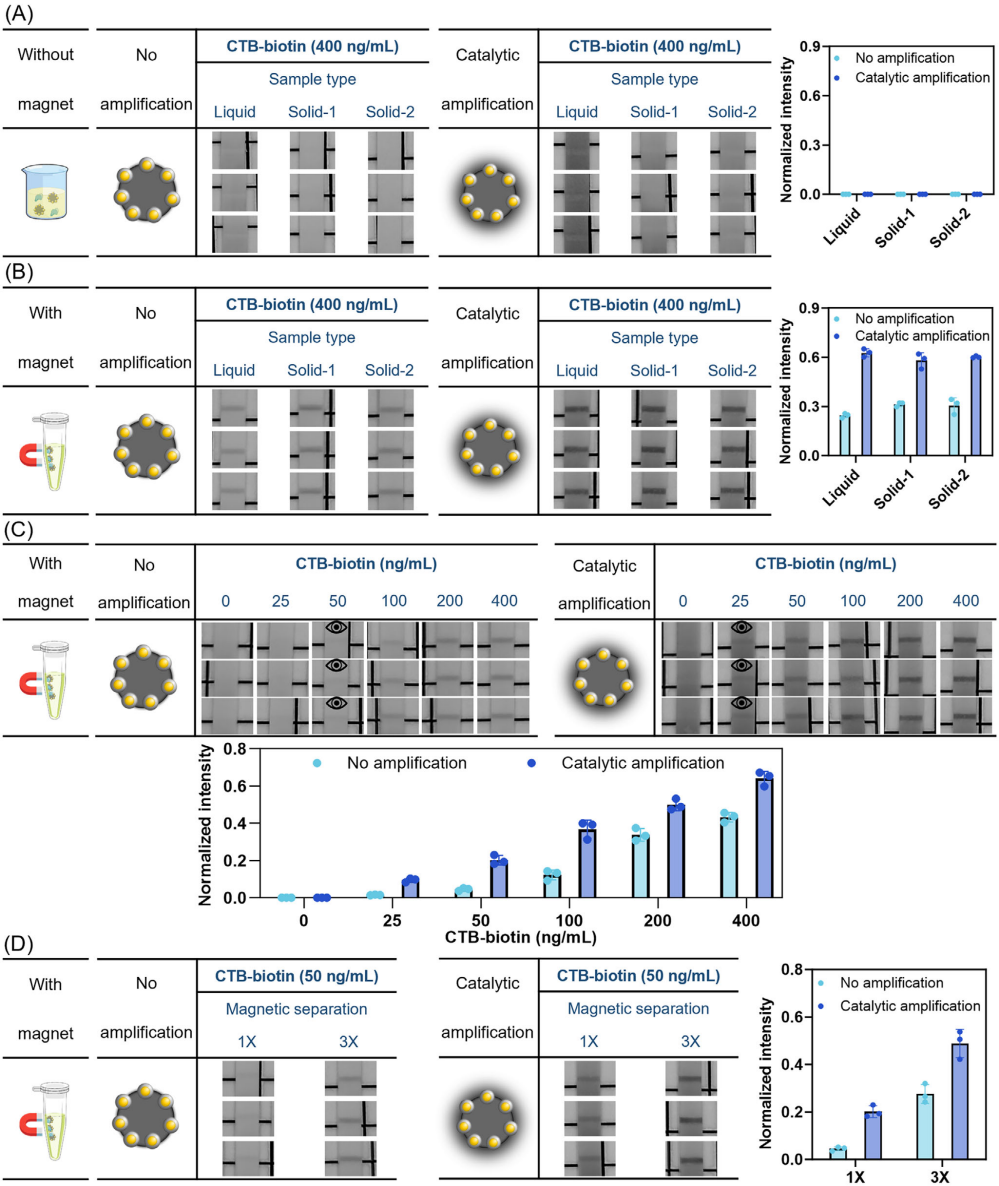
A) Detection of 400 ng mL^−1^ CTB‐biotin with Pt@Fe_3_O_4_@Ab in stools without magnetic separation. Data shown as mean ± S.D., *n* = 3 independent experiments with different batches of nanoparticles. B) Detection of 400 ng mL^−1^ CTB‐biotin with Pt@Fe_3_O_4_@Ab in stools with magnetic separation but no subsequent volumetric concentration. Data shown as mean ± S.D., *n* = 3 independent magnetic separation procedures with different batches of nanoparticles. Post‐catalytic amplification signals show no significant difference among group means by Welch's ANOVA tests, i.e., *p* = 0.9390. C) Detection of different concentrations of CTB‐biotin with Pt@Fe_3_O_4_@Ab in pooled solid stools with magnetic separation, but no subsequent volumetric concentration. Data shown as mean ± S.D., *n* = 3 independent magnetic separation procedures with different batches of nanoparticles. The eye icons represent the visual LOD in these experiments. D) Detection of 50 ng mL^−1^ CTB‐biotin with Pt@Fe_3_O_4_@Ab in pooled solid stools with magnetic separation and either no volumetric concentration (1×) or 3‐fold volumetric concentration (3×) of the sample. Data shown as mean ± S.D., *n* = 3 independent magnetic separation procedures with different batches of nanoparticles. All assays were performed in either liquid stool (single donor) or pooled solid stool (a mixture from 11 donors, 20 µL per donor, diluted 1:20 in DPBS to mimic diarrhea samples). For each magnetic separation procedure, the particles were separated from 180 µL of stool samples and resuspended in 180 µL (B, C, D‐1×) or 60 µL (D‐3×) of running buffer.

As an initial validation, Pt@Fe_3_O_4_ nanoreporters conjugated with a detection antibody were employed to detect the model antigen CTB‐biotin in the running buffer. The Pt@Fe_3_O_4_ system demonstrated effective CTB‐biotin detection both without and with magnetic separation, and the catalytic amplification successfully enhanced the signal level (Figure S11A,B, Supporting Information), consistent with results observed in saliva. Next, we proceeded to evaluate the system using human stool samples. As shown in **Figure** **5**A, when the magnetic separation step was omitted, no detectable signal was observed for CTB‐biotin in human stool samples from various sources (including liquid stools and pooled solid stools from different donors; solid stool samples were mixed and diluted in a 1:20 ratio with DPBS before the assay was performed to mimic diarrhea samples). This result aligned with our observations in pure saliva samples, where the matrix effect posed significant challenges to the consistent flow of nanoreporters through the assay strips. In the stool, we observed large and unidentified solids, which further hindered nanoreporter flow.

We then investigated whether the implementation of magnetic separation could enhance the performance of Pt@Fe_3_O_4_ nanoreporters and facilitate the detection of target antigens in human stool samples. As demonstrated in Figure 5B, the combination of Pt@Fe_3_O_4_ nanoreporters and magnetic separation enabled the successful detection of CTB‐biotin in human stool samples. All test lines appeared clear and distinct, indicating a uniform flow of the particles across the membrane, even when analytes were present in stool samples with complex fluid properties. Notably, the test line intensity remained consistent regardless of sample consistency (whether more solid or liquid) or variations between different batches of patient samples. The well‐defined calibration curves of CTB‐biotin concentration vs test line intensity in Figure 5C demonstrate the excellent antigen concentration‐dependent response of the Pt@Fe_3_O_4_ nanoreporters in a complex stool matrix with magnetic separation. Catalytic amplification reduced the visual LOD to as low as 25 ng mL^−1^. Moreover, we confirmed that increasing the concentration factor could further enhance the signal (Figure 5D). This approach can be particularly useful in compensating for the loss of detection sensitivity caused by diluting solid stool samples in typical LFIA applications, ensuring even sample flow.

The results obtained using the magnetically retrievable Pt@Fe_3_O_4_ nanoreporters in highly complex human stool samples further highlight the potential of Pt@Fe_3_O_4_ combined with magnetic separation in LFIA for samples containing challenging biological matrices. Our system effectively reduced background noise from biological impurities, ensured smooth flow of the nanoreporters, and minimized variability caused by sample heterogeneity, ultimately achieving robust analyte detection in bio‐samples with a high signal‐to‐noise ratio.

## 3 Conclusion

In this study, we developed a magnetically retrievable Pt@Fe_3_O_4_ nanoreporter for LFIAs, consisting of an Fe_3_O_4_ core decorated with PtNCs bound to its surface. The Pt@Fe_3_O_4_ nanoreporters exhibit strong magnetic properties from the Fe_3_O_4_ NPs and high peroxidase‐mimicking activity from the PtNCs. The nanoreporter is robust under various conditions—including drying, long‐term storage, and different sample matrices.

By leveraging the magnetic properties of Pt@Fe_3_O_4_, analytes can be efficiently purified and concentrated from challenging biological samples. Magnetic separation of nanoreporters‐antigen complexes effectively mitigates matrix effects. Without this purification step, the complexity of biological fluids and patient‐to‐patient heterogeneity would otherwise cause high background noise, reduced sensitivity, and poor reproducibility. While the addition of a magnetic separation step increases the complexity of the assay, technologies are available^[^ ^64^ ^]^ to facilitate this process, allowing POC deployment of the tests. In addition to magnetic separation and enrichment, the catalytic activity of PtNCs enables further enhancement of the colorimetric readout through the catalytic oxidation of CN/DAB, leading to dark precipitates on the test line. While practical considerations remain for the use of amplifying reagents such as CN/DAB, given their associated hazards, future work could address this through careful packaging design and integration into lateral flow tests, ensuring safe handling and incorporation of these reagents.

The bio‐samples tested in this study span multiple matrices, including serum, saliva, and, notably, stool, which is rarely analyzed directly without dilution with LFIAs. Such broad applicability suggests potential extension to additional matrices, such as tears or nasopharyngeal swabs, as well as those associated with pathological conditions that can interfere with LFIA performance, including hematuria or hyperlipidemic blood.

Beyond demonstrating applicability across diverse complex bio‐samples, we also conducted experiments using artificial saliva combined with numerical simulations, which revealed that viscosity is a key factor underlying matrix effects and substantially impacts LFIA reproducibility. Although viscosity can strongly influence assay performance, it is often overlooked during product development, leading to variability in real‐world testing. By elucidating the mechanisms through which viscosity affects LFIA performance, our study provides valuable guidance for designing more reliable LFIA devices for practical applications.

Overall, these Pt@Fe_3_O_4_ nanoreporters, enabling magnetic separation, offer a promising approach to improving LFIA performance with complex bio‐samples, effectively addressing challenges that traditional systems often face.

## 4 Experimental Section

### Preparation of PtNCs

Following a modified protocol from Loynachan et al.^[^ ^34^ ^]^ gold nanospheres (5 nm) were diluted into 10 mL of Milli‐Q water to a final concentration of 0.017 mg mL^−1^, serving as seeds. To this solution, 200 µL of 200 mg mL^−1^ PVP (10 kDa) was added and stirred for 1 h to allow the polymer to coat and stabilize the gold seeds. The resultant solution was then heated to 80 °C. Subsequently, 400 µL of 100 mg mL^−1^ L‐ascorbic acid was introduced to the mixture, followed by 400 µL of 100 mm chloroplatinic acid hydrate. The solution was mixed thoroughly and immediately incubated at 80 °C while stirring at 300 rpm. After a 45‐min reaction, the synthesized PtNCs were rapidly cooled in an ice bath and stored at 4 °C for further use.

### Preparation and Characterization of Pt@Fe_3_O_4_

Pt@Fe_3_O_4_ was prepared by adding 25 µL of 50 mg mL^−1^ Fe_3_O_4_ NPs to 500 µL of PtNCs (synthesized as described above), followed by immediate vortexing and continuous shaking at 500 rpm overnight. The product was magnetically washed twice with Milli‐Q water, redispersed in 100 µL of Milli‐Q water, and stored at 4 °C for further use. The hydrodynamic size and zeta potential of nanoparticles were characterized using DLS (Zeta Sizer Nanoseries, Malvern Instruments Ltd., UK). For electron microscopy characterization, the nanoparticle solution was deposited onto carbon‐coated copper grids (Agar Scientific, UK) and allowed to dry. The procedures followed a previously established protocol.^[^ ^34^ ^]^ Specifically, TEM imaging was carried out using a JEOL 2100F microscope (JEOL, Ltd., Japan) at 200 kV, fitted with a Gatan Orius SC 1000 SD camera (Gatan, Inc., USA). Elemental composition mapping of the nanoparticles was performed through EDS in STEM mode, using the JEOL 2100F equipped with a Gatan annular bright field detector, a Gatan high‐angle annular dark field detector, and an EDS detector (Oxford Instruments, UK). The Pt and Fe content in Pt@Fe_3_O_4_ was quantified using an iCAP6000 ICP spectrometer (Thermo Fisher Scientific, USA). The samples were prepared by digesting the nanoparticles in aqua regia, followed by dilution with Milli‐Q water. Gradient‐diluted Pt and Fe ICP standard solutions (Thermo Fisher Scientific, USA) were used for calibration. The crystal structure of Pt@Fe_3_O_4_ was analyzed with a D2 Phaser X‐ray diffractometer ((Bruker, Germany), with a scan type of coupled TwoTheta/Theta, a scan mode of continuous PSD fast, and a scan speed of 0.5 s per step. Magnetization curves of Fe_3_O_4_ NPs and Pt@Fe_3_O_4_ were obtained using an MPMS3‐SQUID‐VSM magnetometer (Quantum Design International, USA).

### Detection Antibody Conjugation on Pt@Fe_3_O_4_ Nanoreporters

To conjugate detection antibodies onto the Pt@Fe_3_O_4_ surface, 50 µL of Pt@Fe_3_O_4_ stock was mixed with 50 µL of Milli‐Q water, 10 µL of HEPES buffer (pH 8.2, 100 mm), and 10 µL of DPBS containing 3.125 µm detection antibodies (equivalent to 500 antibodies per nanoparticle). The mixture was incubated for 3 h on a shaker at 500 rpm. Next, 100 µL of 2 wt.% PVP (10 kDa) in DPBS was added to the mixture to block the antibody‐conjugated Pt@Fe_3_O_4_. After an additional 1‐h incubation, the products were magnetically washed three times with 2 wt.% PVP (10 kDa) in DPBS and then redispersed in 100 µL of 2 wt.% PVP (10 kDa) in DPBS.

### Detection of N‐Protein in Human Saliva using Pt@Fe_3_O_4_ Nanoreporters

All saliva samples used in this study were obtained from commercial sources (Lee BioSolutions, St. Louis, USA) and provided as pooled, fully anonymized products without individual‐level information. The provider obtained the samples under informed consent.

Without magnetic separation: 1.06 µL of Pt@Fe_3_O_4_ stock (conjugated with antibody 40143‐MM08) was added to 63 µL of human saliva or running buffer (fetal bovine serum (FBS) with 1 wt.% PVP, 10 kDa) containing different concentrations of N‐protein. After a 40‐min incubation, lateral flow strips with a 40143‐R001 antibody test line were dipped into a non‐binding Corning 96‐well plate, with each well containing 64 µL of different samples. After allowing the solution to wick through the strips for 10 min, the strips were transferred to another well containing 100 µL of running buffer and incubated for an additional 10 min. Finally, the strips were immersed in 500 µL of amplification solution (50 µL CN/DAB kit, 250 µL stable peroxide substrate buffer, and 200 µL 30 wt.% H_2_O_2_) for 10 min. The strips were washed in Milli‐Q water for 5 s to quench the amplification reaction.

With magnetic separation and either no volumetric concentration (1) or 5‐fold volumetric concentration (5×): 3.26 µL (or 1.14 µL for 5‐fold volumetric concentration) of Pt@Fe_3_O_4_ stock was added to 200 µL (or 350 µL for 5‐fold volumetric concentration) of human saliva or running buffer containing different concentrations of N‐protein. After a 20‐min incubation, the Pt@Fe_3_O_4_‐antigen complexes were collected using a DynaMag‐2 magnetic rack and resuspended in 200 µL (or 70 µL for 5‐fold volumetric concentration) of running buffer. Lateral flow strips with a 40143‐R001 antibody test line were then dipped into a non‐binding Corning 96‐well plate, with each well containing 65 µL of different samples. After allowing the solution to wick through the strips for 10 min, the strips were moved to another well containing 100 µL of running buffer and wick for an additional 10 min. Finally, the strips were immersed in 500 µL of amplification solution (50 µL CN/DAB kit, 250 µL stable peroxide substrate buffer, and 200 µL of 30 wt.% H_2_O_2_) for 10 min. The strips were washed in Milli‐Q water for 5 s to quench the amplification reaction.

### Detection of CTB‐Biotin in Human Stool Samples using Pt@Fe_3_O_4_ Nanoreporters

Ethical approval for stool collection was granted by an NHS Research Ethics Committee, reference 17/LO/1420, as part of work on the development of rapid diagnostics. This approval covers the use of routinely collected samples processed by the John Radcliffe Hospital Microbiology laboratory, and due to be discarded at the end of routine clinical workflows, for research into the development and evaluation of diagnostic assays, without patient‐level consent. The collection was performed by trained personnel, including biomedical scientists, apprentices, and medical laboratory assistants from OUH Microbiology. Samples were stored at 4 °C for up to two weeks prior to use.

For experiments, a liquid stool sample from a single donor was used directly. For pooled solid samples, stool from 11 different donors (20 µL per donor, collected using a Pasteur microloop) was combined in 4.4 mL of DPBS and vigorously vortexed to mimic diarrhea samples.

Without magnetic separation: 0.90 µL of Pt@Fe_3_O_4_ stock (conjugated with antibody F5J, MA5‐18188) was added to 54 µL of stool sample or running buffer (FBS with 1 wt.% PVP, 10 kDa) containing different concentrations of CTB‐biotin. After a 40‐min incubation, lateral flow strips with a poly‐streptavidin test line were dipped into a non‐binding Corning 96‐well plate, with each well containing 54.9 µL of different samples. After allowing the solution to wick through the strips for 10 min, the strips were transferred to another well containing 100 µL of running buffer and incubated for an additional 10 min. Finally, the strips were immersed in 500 µL of amplification solution (50 µL CN/DAB kit, 250 µL stable peroxide substrate buffer, and 200 µL 30 wt.% H_2_O_2_) for 10 min. The strips were washed in Milli‐Q water for 5 s to quench the amplification reaction.

With magnetic separation and either no volumetric concentration (1×) or 3‐fold volumetric concentration (3×): 2.93 µL (or 0.98 µL for 3‐fold volumetric concentration) of Pt@Fe_3_O_4_ stock was added to 180 µL of stool sample or running buffer containing different concentrations of CTB‐biotin. After a 20‐min incubation, the Pt@Fe_3_O_4_‐antigen complexes were collected using a DynaMag‐2 magnetic rack and resuspended in 180 µL (or 60 µL for 3‐fold volumetric concentration) of running buffer. Lateral flow strips with a poly‐streptavidin test line were then dipped into a non‐binding Corning 96‐well plate, with each well containing 55 µL of different samples. After allowing the solution to wick through the strips for 10 min, the strips were moved to another well containing 100 µL of running buffer and run for an additional 10 min. Finally, the strips were immersed in 500 µL of amplification solution (50 µL CN/DAB kit, 250 µL stable peroxide substrate buffer, and 200 µL of 30 wt.% H_2_O_2_) for 10 min. The strips were washed in Milli‐Q water for 5 s to quench the amplification reaction.

### Image Analysis

For lateral flow strip experiments, two images were captured for the strips: one immediately after removal from the 10‐min running buffer wicking (no amplification), and the other immediately after the Milli‐Q water washing (catalytic amplification).

Images of lateral flow strips were captured using an Apple iPhone 14 and analyzed with ImageJ 1.54f software. The images were first converted into 8‐bit format. A rectangular region of interest of 80 × 200 pixels was drawn around the test line using the gel analysis function. The peak area of the plotted lanes was then used to determine the signal intensity of the test line. The intensity of the test line was normalized against the intensity of the background grid to mitigate potential interference caused by variations in imaging conditions, such as lighting. Specifically, the normalized intensity was calculated as the ratio of the intensity of the test line to the intensity of the background grid, i.e., normalized intensity = peak area of the test line/peak area of the background grid.

### Statistics

All statistical analyses were performed using GraphPad Prism 10.1.2. Group comparisons were conducted using Welch's ANOVA tests, which are appropriate for datasets with unequal variances among groups. The resulting *p*‐values are reported in the corresponding figure captions.

Data are presented as mean ± standard deviation (SD) unless otherwise specified. The sample size (*n*) for each experiment is provided in the corresponding figure captions.

Normalization of the strip test line intensity is described under the subhead “Image Analysis” of the Experimental Section.

The LOD for all antigen detection experiments was determined based on the minimum visible colorimetric signals observed under experimental settings.

All other experimental details are shown in the Supporting Information.

## Conflict of Interest

C.J.S. and A.S. have consulted for a company related to nanomaterials and assays for biosensing; they have filed patent applications relating to nanomaterials for biosensing. M.M.S. has invested in, consulted for (or is on scientific advisory boards or boards of directors), and conducted sponsored research funded by companies related to the biomaterials field; has filed patent applications related to nanomaterials and assays for biosensing; and has co‐founded companies in the diagnostics field. The rest of the authors declare no conflict of interest.

## Author Contributions

Y.C. led the project under the supervision of M.M.S. and performed all experiments. L.P. provided extensive support throughout the project, contributing to discussions on optimization and overall project development. L.P. and A.C. conceived the initial project idea. C.J.S., A.C., and A.S. provided technical training related to LFIA experiments. A.S. assisted in the preparation of the schematic illustrations. K.L. and T.G. conducted the calculation and modeling work on nanoreporter transport and reaction kinetics. A.V. assisted with the use of patient stool samples. Y.C. drafted the manuscript with input from all authors. All authors contributed to the interpretation of results and reviewed and commented on the manuscript.

## Supporting information



Supporting Information

## Data Availability

The data that support the findings of this study are available from the corresponding author upon reasonable request.
