# Supplementary material for: Magnetically Retrievable Platinum Nanoreporters for Efficient Lateral Flow Immunoassay in Complex Bio‐Samples
Source: Small. 2025 Nov 28;22(2):e06622. doi: 10.1002/smll.202506622 (PMC12781628; doi:10.1002/smll.202506622)
Supplement: Supplementary file 1 — Supporting Information [file SMLL-22-e06622-s001.docx]

Magnetically Retrievable Platinum Nanoreporters for Efficient Lateral Flow Immunoassay in Complex Bio-samples

Yuxi Cheng^1, 2^, Luca Panariello^1, 2, 3^, Adam Creamer^1, 2^, Christy J. Sadler^1, 2^, André Shamsabadi^1, 2^, Kathleen Lupien,^4^ Ali Vaughan,^5, 6^ Thomas Gervais,^4^ Molly M. Stevens^1, 2, 3, *^

1 Department of Materials, Department of Bioengineering, and Institute of Biomedical Engineering, Imperial College London, London SW7 2AZ, U.K.

2 Department of Physiology, Anatomy and Genetics, Department of Engineering Science, and Kavli Institute for Nanoscience Discovery, University of Oxford, Oxford OX1 3QU, U.K.

3 Department of Medical Biochemistry and Biophysics, Karolinska Institutet, Stockholm 171 11, Sweden.

4 Department of Engineering Physics, Polytechnique Montréal, Montréal H3T 1J4, Canada

5 Nuffield Department of Medicine, University of Oxford, Oxford OX3 9DU, U.K.

6 NIHR Oxford Biomedical Research Centre, University of Oxford, Oxford OX3 9DU, U.K.

^*^Corresponding author. E-mail: [molly.stevens@dpag.ox.ac.uk](mailto:molly.stevens@dpag.ox.ac.uk)

**Supporting Information**

**Experimental Section**

**Materials**

5 nm gold nanospheres were purchased from nanoComposix (San Diego, USA). Polyvinylpyrrolidone (PVP, 10 kDa), L-ascorbic acid, chloroplatinic acid hydrate, sodium carboxymethylcellulose (SCMC, 90 kDa), D-sorbitol and normal human serum were purchased from Sigma-Aldrich (St. Louis, USA). Magnetic silica beads (Fe_3_O_4_ nanoparticles, Fe_3_O_4_ NPs) were purchased from Alpha Nanotech Inc. (Vancouver, Canada). HER2 antigen (amine-labelled biotinylated, 10004-H08H-B), SARS-CoV-2 nucleocapsid antigen (40588-V08B), SARS-CoV/SARS-CoV-2 nucleocapsid antibody (mouse mAb, 40143-MM08), and SARS-CoV/SARS-CoV-2 nucleocapsid antibody (rabbit mAb, 40143-R001) were purchased from Sino Biological (Beijing, China). Trastuzumab was purchased from Biosynth Carbosynth (Berkshire, UK). Poly-streptavidin lateral flow strips were obtained from Mologic Ltd. (Bedfordshire, UK). Unisart® CN95 nitrocellulose membrane was purchased from Sartorius (Göttingen, Germany). KN-V1060.44 backing card was purchased from Kenosha (Amsterdam, Netherlands). KN-222-20 absorbent pad was purchased from Ahlstrom (Helsinki, Finland). Cholera toxin beta monoclonal antibody F5J (MA5-18188), Cholera toxin subunit B (recombinant, biotin conjugated, C34779), Dulbecco's phosphate-buffered saline (DPBS, 1×), fetal bovine serum (FBS), Pierce CN/DAB substrate kit and Pierce 1-Step TMB substrates were purchased from Thermo Fisher Scientific (Waltham, USA). Normal human saliva was purchased from Lee Biosolutions (St. Louis, USA). TMB X-tra was purchased from 2B Scientific (Camarillo, USA).

**Evaluation of peroxidase-mimicking activity of Pt@Fe_3_O_4_ nanoreporters**

The peroxidase‐mimicking activities of Pt@Fe₃O₄ nanozymes were assessed by determining their specific activity (*SA*) and catalytic efficiency (*K_cat_*) through the colorimetric oxidation reaction of TMB. All experiments and calculations were performed following previously reported procedures,^1, 2^ unless otherwise specified. Assays were conducted in 0.2 M NaOAc/HOAc buffer (pH 4.0). Absorbance at 653 nm (*Abs_653nm_*) was recorded in non-binding 96-well plates using a SpectraMax M5 microplate reader (Molecular Devices, USA). Reported absorbance values were path-length corrected to *l* = 1.0 cm.

*Determination of specific activity, SA*. One unit of nanozyme activity (*U*) was defined as the amount of nanozyme that converts 1 μmol of substrate per minute. The *SA* was expressed as activity units per milligram of nanozyme. For a typical assay, Pt@Fe₃O₄ samples with varying Pt(0) contents (1.5, 0.75, 0.375, 0.1875, 0.15, 0.09, 0.075, and 0 ng, as determined by previous ICP-MS tests) were incubated with 0.8 mM TMB and 2.0 M H₂O₂ in 200 μL of acetate buffer. Parallel samples prepared with Milli-Q water instead of H₂O₂ served as blanks for background subtraction. Absorbance changes at *λ*=653 nm were continuously recorded at 15 s intervals for 10 min at room temperature. Initial reaction rates were obtained from the linear portion of the “*Abs653nm versus time*” curves (first 30 s). The nanozyme activity (*b_nanozyme_,* Units) was calculated using: *V* / (*ε* × *l*) × (Δ*A* / Δ*t*), where *V* is the reaction volume (200 μL), *ε* is the molar absorption coefficient of oxidized TMB at 653 nm (39,000 M⁻¹·cm⁻¹), *l* is the optical path length (1 cm), and Δ*A*/Δ*t* is the initial rate of absorbance change (min⁻¹). Because *b_nanozyme_* scales linearly with nanozyme mass, *SA* (U·mg⁻¹) was determined from the slope of a linear regression of *b_nanozyme_ versus* Pt mass in the samples.

*Determination of catalytic efficiency, K_cat_*. For kinetic measurements, Pt@Fe₃O₄ nanozymes were used at a particle concentration of 1.67 × 10⁻¹⁶ M, corresponding to 0.5 ng Pt(0) as quantified previously by ICP-MS and NanoFCM. Reaction mixtures contained 200 μL of acetate buffer, nanozymes, 2.0 M H₂O₂, and TMB at varying concentrations (1.6, 0.8, 0.6, 0.5, 0.4, 0.2, 0.1, and 0 mM). Parallel samples prepared with Milli-Q water instead of H₂O₂ served as blanks for background subtraction. Abs_653nm_ was continuously recorded at 15 s intervals for 10 min at room temperature. Initial reaction rates were obtained from the linear portion of the “*Abs_653nm_ versus time*” curves (first 30 s), and initial velocities (*ν*, M·s⁻¹) were calculated as: *ν* = (Δ*A* / Δ*t*) / (*ε* × *l*), where the definitions of the parameters are identical to those used for the calculation of *SA*. The dependence of *ν* on TMB concentration was then fitted to the Michaelis–Menten equation: *ν* = *V_max_* × *[S]* / (*K_m_* + *[S]*), where *[S]* is the TMB concentration, *K_m_* is the Michaelis constant, and *V_max_* is the maximum velocity. Nonlinear regression yielded *K_m_* and *V_max_*. The catalytic efficiency (*K_cat_*) was calculated from: *K_cat_* = *V_max_* / *[E]*, where *[E]* is the nanozyme concentration (1.67 × 10⁻¹⁶ M). Surface area–normalized catalytic efficiency (*K_cat_-specific*) was not reported due to the heterogeneous surface structure of Pt@Fe₃O₄, which prevents reliable estimation of accessible surface area.

**Preparation of capture antibody labelled lateral flow strips**

The lateral flow strips consisted of three components: CN95 nitrocellulose membrane, absorbent pad, and backing card. The test line was printed at a height of 10 mm from the bottom of the nitrocellulose membrane using 0.5 mg/mL capture antibody solutions with a BioDot Spotter AD-BioJet (Irvine, USA). The nitrocellulose membrane was then dried overnight at 37 °C. Subsequently, the lateral flow strips were assembled by attaching the nitrocellulose membrane and absorbent pad to the backing card. A BioDot cutter (Irvine, USA) was used to cut the assembled membranes into 3 mm wide strips.

**Detection of HER2-biotin in human serum using Pt@Fe_3_O_4_ nanoreporters**

All serum samples used in this study were obtained from commercial sources (Sigma-Aldrich, St. Louis, USA) and provided as pooled, fully anonymized products without individual level information. The provider obtained the samples under informed consent.

Without magnetic separation: 1.06 μL of Pt@Fe_3_O_4_ stock (conjugated with Trastuzumab) was added to 63 μL of human serum or running buffer (FBS with 1 wt% PVP, 10 kDa) containing varying concentrations of HER2-biotin. After a 40-minute incubation, lateral flow strips with a poly-streptavidin test line were dipped into a non-binding Corning® 96-well plate, with each well containing 64 μL of different samples. After allowing the solution to wick through the strips for 10 minutes, the strips were transferred to another well containing 100 μL of running buffer and wick for an additional 10 minutes.

With magnetic separation and 20-fold volumetric concentration (20×): 1.14 μL of Pt@Fe_3_O_4_ stock was added to 1400 μL of human serum or running buffer containing varying concentrations of HER2-biotin. After a 20-minute incubation, the Pt@Fe_3_O_4_-HER2-biotin complexes were collected using a DynaMag-2 magnetic rack and resuspended in 70 μL of running buffer. The corresponding lateral flow strips were then dipped into a non-binding Corning® 96-well plate, with each well containing 65 μL of different samples. After allowing the solution to run through the strips for 10 minutes, the strips were moved to another well containing 100 μL of running buffer and wick for an additional 10 minutes.

**Detection of N-protein in artificial saliva using Pt@Fe_3_O_4_ nanoreporters**

Artificial saliva was prepared by dissolving 3 wt% D-sorbitol and 0-1.25 wt% sodium carboxymethylcellulose (SCMC, 90 kDa) in DPBS.

Without magnetic separation: 1.06 μL of Pt@Fe_3_O_4_ stock (conjugated with antibody 40143-MM08) was added to 63 μL of artificial saliva containing 20 nM N-protein. After a 40-minute incubation, lateral flow strips with a 40143-R001 antibody test line were dipped into a non-binding Corning® 96-well plate, with each well containing 64 μL of different samples. After allowing the solution to wick through the strips for 10 minutes, the strips were transferred to another well containing 100 μL of running buffer (FBS with 1 wt% PVP, 10 kDa) and incubated for an additional 10 minutes. Finally, the strips were immersed in 500 μL of amplification solution (50 μL CN/DAB kit, 250 μL stable peroxide substrate buffer, and 200 μL 30 wt% H₂O₂) for 10 minutes. The strips were washed in Milli-Q water for 5 s to quench the amplification reaction.

With magnetic separation but no subsequent volumetric concentration (1×): 3.26 μL of Pt@Fe_3_O_4_ stock was added to 200 μL of artificial saliva containing 20 nM N-protein. After a 20-minute incubation, the Pt@Fe_3_O_4_-antigen complexes were collected using an a DynaMag-2 magnetic rack and resuspended in 200 μL of running buffer. Lateral flow strips with a 40143-R001 antibody test line were then dipped into a non-binding Corning® 96-well plate, with each well containing 65 μL of different samples. After allowing the solution to wick through the strips for 10 minutes, the strips were moved to another well containing 100 μL of running buffer and wick for an additional 10 minutes. Finally, the strips were immersed in 500 μL of amplification solution (50 μL CN/DAB kit, 250 μL stable peroxide substrate buffer, and 200 μL of 30 wt% H₂O₂) for 10 minutes. The strips were washed in Milli-Q water for 5 s to quench the amplification reaction.

**Modeling calculations**

***Table S1.*** *Model parameters, variable names, and corresponding values and units used in the transport and reaction kinetics simulations.*

|  | Variable | Value | Units |
| --- | --- | --- | --- |
| Strip width | *w* | 3 | mm |
| Strip thickness | *h* | 155 | μm |
| Test line width | *e* | 0.8 | mm |
| Total strip length | *L* | 40 | mm |
| Height of test line | $l$ | 10 | mm |
| Pt@Fe_3_O_4_ particle radius | $R_{p}$ | 150 | nm |
| Concentration of antigen in the sample | $\left[ Ag \right]_{0}$ | 20 | nM |
| Concentration of Pt@Fe_3_O_4_ | $C_{NP}$ | 1.36 | pM |
| Equivalence of detection antibody $Ab_{p}$ on Pt@Fe_3_O_4_ | - | 500/nanoparticle | N/A |
| Concentration of capture antibody ($Ab_{c}$) | $C_{Ab_{c}}$ | 4.03 ^(i)^ | μM |
| Detection antibody $k_{on}$ | $k_{{on}_{p}}$ | 7.51 × 10^5^ ^(ii)^ | M^−1^⋅s^−1^ |
| Detection antibody $k_{off}$ | $k_{{off}_{p}}$ | 1.79 × 10^-5^ ^(ii)^ | s^−1^ |
| Detection antibody $k_{d}$ | $k_{d_{p}}$ | 0.024 ^(ii)^ | nM |
| Capture antibody $k_{on}$ | $k_{{on}_{c}}$ | 1.21 × 10^6^ ^(iii)^ | M^−1^⋅s^−1^ |
| Capture antibody $k_{off}$ | $k_{{off}_{c}}$ | 1.4 × 10^-5^ ^(iii)^ | s^−1^ |
| Capture antibody $k_{d}$ | $k_{d_{c}}$ | 0.011 ^(iii)^ | nM |
| Density of Pt@Fe_3_O_4_ | $\rho_{p}$ | 4680 ^(iv)^ | kg⋅m^−3^ |
| Lowest sample viscosity | - | 1.1 ^(v)^ | mPa⋅s |
| Highest sample viscosity | - | 6.6 ^(vi)^ | mPa⋅s |

^(i)^ This value was determined from the molar amount of capture antibody used to print a single test line, together with the test line’s volume.

^(ii)^ The affinity data of the detection antibody was obtained from the manufacturer.

^(iii)^ The affinity data of the capture antibody was obtained from the literature.^3^

^(iv)^ This value was calculated from the ICP-MS measured elemental composition of the nanoreporter (Pt, Fe, O, and Si), combined with the bulk densities of platinum, iron oxide, and silica.

^(v)^ This value was obtained experimentally using a rheometer to measure the viscosity of the artificial saliva containing 0 wt% SCMC at a shear rate of 100 s⁻¹.

^(vi)^ This value was obtained experimentally using a rheometer to measure the viscosity of artificial saliva containing 1.25 wt% SCMC at a shear rate of 100 s⁻¹.

Diffusion time

The diffusion coefficient of Pt@Fe₃O₄ nanoreporters was estimated using the Stokes-Einstein equation:

$$\begin{aligned} D_{NP}=\frac{k_{b}T}{6\pi\eta r}\#\left( S1 \right) \end{aligned}$$

Assuming:

- Temperature = 293 K
- Particle radius r = 150 nm
- *k_b_*​=1.38 × 10^−23^ J/K

The estimated diffusion coefficient is $D_{NP}$ = 1.30 × 10^-12^ m²/s for the lowest viscosity (1.1 mPa⋅s), and 2.17 × 10^-13^ m²/s for the highest viscosity (6.6 mPa⋅s). Using test line length $\Delta x=e=0.8 mm$: the characteristic diffusion time $t_{d}=\frac{{\Delta x}^{2}}{D}=$6 days (for $\eta= 1.1 \mathrm{mPa}\cdot s$) and 34 days (for $\eta= 6.6 \mathrm{mPa}\cdot s$).

Reaction time

For the capture antibody reaction (which is independent of viscosity):

$$\begin{aligned} t_{r}=\frac{1}{k_{{on}_{c}}C_{Ab_{c}}}\#\left( S2 \right) \end{aligned}$$

$$=0.205 s$$

Convection time

Assuming capillary flow through porous media follows the Washburn equation, the position of the fluid front in the strip over time is given by:

$$\begin{aligned} x= \sqrt{\frac{r_{p}\sigma\cos\left( \theta\right)t}{2\eta}}=\sqrt{\frac{D_{w}}{\eta}t}= \sqrt{K_{w}t}\#\left( S3 \right) \end{aligned}$$

where $K_{w}$ is Washburn constant and $D_{w}$ is $K_{w}\eta$. Given that it takes 90 seconds ($t_{fill}$) for water to reach the end of the 40 mm nitrocellulose membrane ($x=L=40 mm$): $K_{w}=1.778\times{10}^{-5} m^{2}/s$and $D_{w}=1.778\times{10}^{-8} m^{2}\cdot Pa$ (given $\eta_{w}= 1.0 \mathrm{mPa}\cdot s$).

The shortest convection time occurs when the fluid front reaches the test line, as the flow velocity decreases over time. By differentiating equation (S3), the instantaneous velocity is obtained as:

$$\begin{aligned} \dot{x}= \frac{D_{w}}{2\eta x}\#\left( S4 \right) \end{aligned}$$

At the test line ($x=l=10 mm$), the instantaneous velocity $\dot{x}$: 0.81 mm/s (lowest *η*), or 0.13 mm/s (highest *η*). The corresponding convection time through the test line: $t_{c}=\frac{e}{\dot{x}(l)}=$ 0.99 s (lowest *η*), or 5.9 s (highest *η*).

As the fluid continues to travel past the test line, the velocity decreases further and becomes constant once the sample reaches the absorbent pad, at time $t_{fill}$. A more realistic estimate of convection time can therefore be obtained by using the average velocity throughout the experiment. Considering both a Washburn regime before reaching the absorbent pad and a constant velocity regime after, the average convection velocity is obtained as:

$$\bar{v_{c}}=\frac{1}{t_{tot}}\left( \int_{0}^{t_{fill}} v\left( t \right)dt+v\left( t_{tot}-t_{fill} \right) \right)$$

$$\begin{aligned} =\frac{1}{t_{tot}}\cdot\left( \frac{D_{w}t_{tot}}{2\eta L}+\frac{L}{2} \right)\#\left( S5 \right) \end{aligned}$$

The resulting average convection time is: $\left\langle t_{c} \right\rangle=\frac{e}{\bar{v_{c}}}=$ 3.40 s (lowest *η*), 11.94 s (highest *η*).

Sedimentation time

The sedimentation speed of Pt@Fe_3_O_4_ nanoreporters in solution is given by:

$$\begin{aligned} v_{0}=\frac{2\Delta\rho g{R_{p}}^{2}}{9\eta}\#\left( S6 \right) \end{aligned}$$

Thus, $v_{0}$ ranges from $0.164 \mu m/s$ (lowest *η*) to 0.027 $\mu m/s$ (highest *η*).

The resulting sedimentation time is: $t_{s}=\frac{e}{v_{0}}=$4878 s (lowest *η*), 29630 s (highest *η*).

These results confirm that convection is the dominant transport mechanism in the system, with both diffusion and sedimentation playing negligible roles under the experimental conditions considered.

Average diffusion time for a nanoparticle to reach an antibody

The average distance a particle must travel before reaching an antibody is:

$$\begin{aligned} \lambda\approx\left( \frac{1}{C_{Ab_{c}}N_{A}} \right)^{\frac{1}{3}}\#\left( S7 \right) \end{aligned}$$

Using a characteristic length scale of *λ* = 74.4 nm, the diffusion time required for a particle to reach an antibody is estimated to be approximately 4.25 ms and 25.4 ms under the lowest and highest viscosity conditions, respectively.


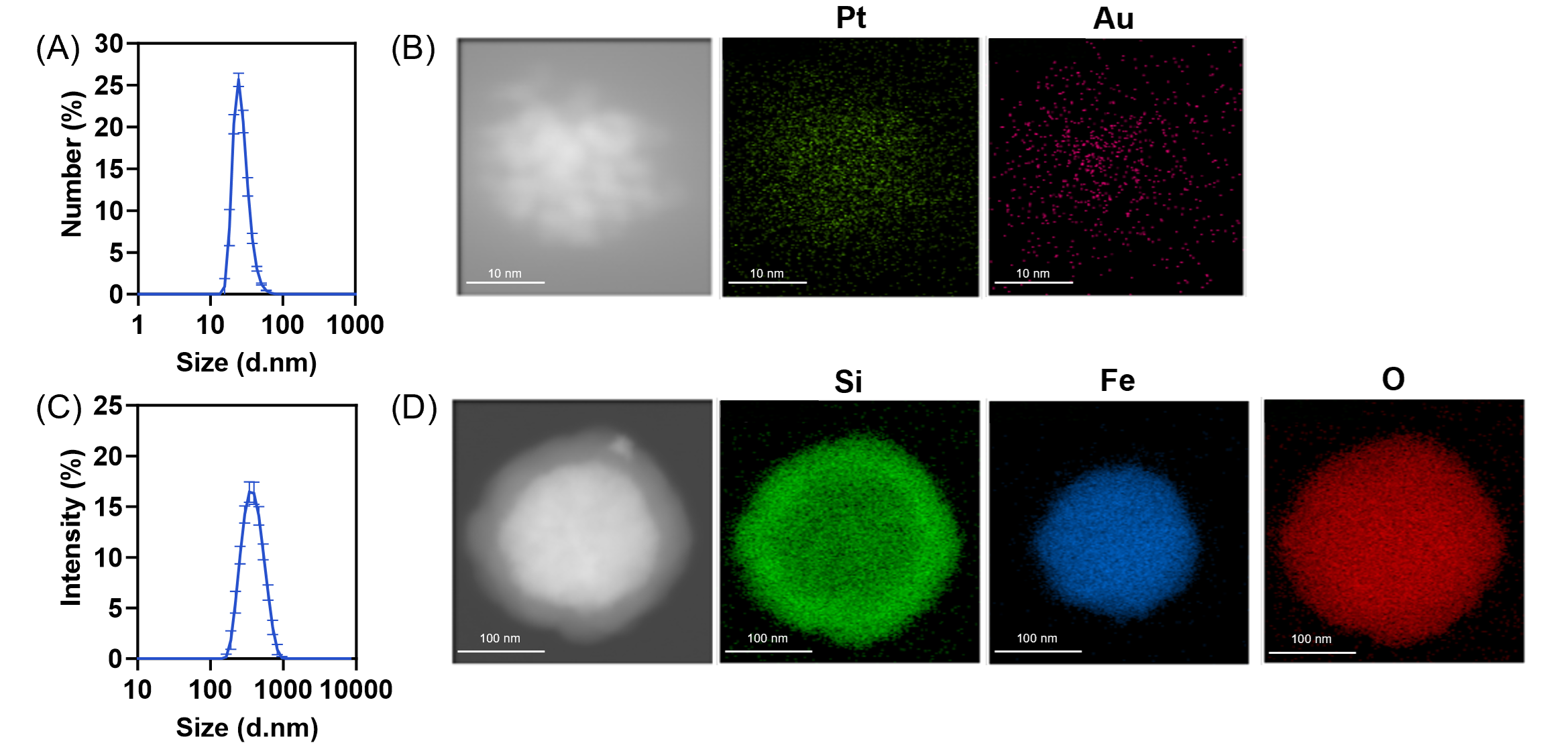


***Figure S1* *(A)*** *Hydrodynamic diameter distribution of PtNCs by DLS. Data shown as mean ± S.D. (n = 3 separate measurements).* ***(B)*** *STEM imaging and EDS mapping showing the elemental composition of PtNCs (scale bar, 10 nm).* ***(C)*** *Hydrodynamic diameter distribution of Fe₃O₄ NPs by DLS. Data shown as mean ± S.D. (n = 3 separate measurements).* ***(D)*** *STEM imaging and EDS mapping showing the elemental composition of Fe₃O₄ NPs (scale bar, 100 nm).*

***
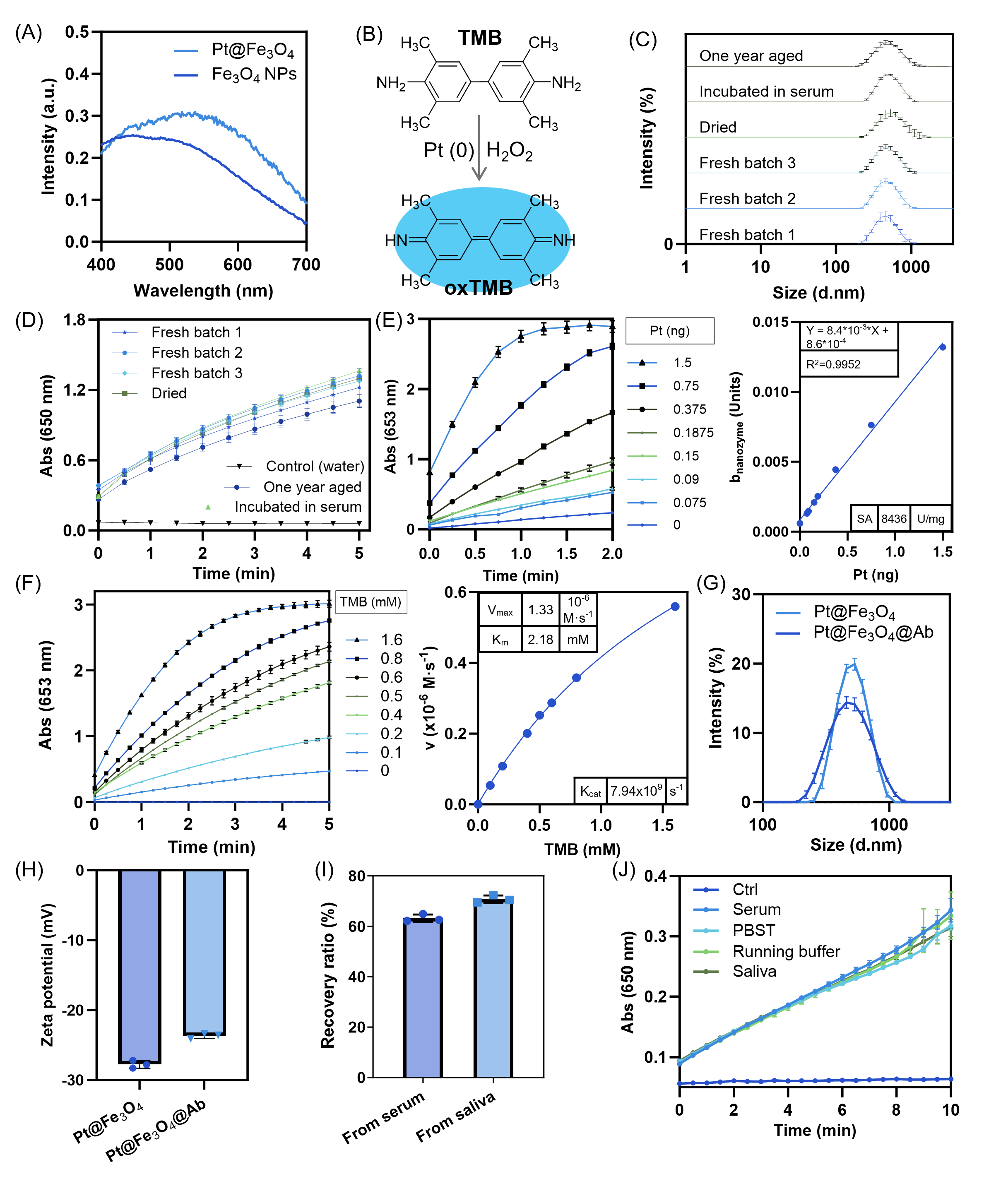
***

***Figure S2 (A)*** *UV–vis absorption spectra of Pt@Fe₃O₄ and Fe₃O₄ NPs at the same concentration. Data shown as a mean of n = 3 separate measurements.* ***(B)*** *Schematic representation of the oxidation reaction of TMB catalyzed by Pt(0) with the presence of H_2_O_2_ to generate blue products.* ***(C)*** *Hydrodynamic diameter distribution of Pt@Fe₃O₄ measured by DLS treated in different conditions (“Fresh batch 1-3” refers to freshly synthesized Pt@Fe₃O₄ in three independent batches, “Dried” refers to Pt@Fe₃O₄ dried into powder and then redispersed in water, “Incubated in serum” refers to Pt@Fe₃O₄ incubated in human serum at 37 °C for 6 hours, magnetically separated, washed, and then redispersed in water, and “One year aged” refers to Pt@Fe₃O₄ stored at 4 °C for 1 year). Data shown as mean ± S.D. (n = 3 separate measurements).* ***(D)****Time-resolved evolution of the absorbance at 650 nm caused by the oxidation of TMB in the presence of H_2_O_2_ catalyzed by Pt@Fe₃O₄ treated in different conditions (same as in* ***Figure S2C****).* *Water was used as the control condition. Data shown as mean ± S.D., n = 3 separate plate wells, with a final particle concentration of 0.2 pM.* ***(E)*** *Time-resolved evolution of the absorbance at 653 nm caused by the oxidation of TMB in the presence of H_2_O_2_ catalyzed by Pt@Fe₃O₄* *with different amount of Pt (ng), and the specific activity (SA, 8436 U/mg) obtained from the amount of Pt nanozyme and the initial slope of the TMB reaction kinetic curves. Data shown as mean ± S.D., n = 3 separate plate wells.* ***(F)*** *Time-resolved evolution of the absorbance at 653 nm caused by the oxidation of different concentrations of TMB (mM) in the presence of H_2_O_2_ catalyzed by Pt@Fe₃O₄, and the catalytic efficiency (K_cat_, 7.94 × 10^9^ s^-1^) obtained from the initial reaction velocity and the Michaelis-Menten equation. Data shown as mean ± S.D., n = 3 separate plate wells.* ***(G)*** *Hydrodynamic diameter distribution of Pt@Fe₃O₄@Ab versus Pt@Fe₃O₄ measured by DLS. Data shown as mean ± S.D. (n = 3 separate measurements).* ***(H)*** *Zeta potential of Pt@Fe₃O₄@Ab versus Pt@Fe₃O₄. Data shown as mean ± S.D. (n = 3 separate measurements).* ***(I)*** *Recovery ratio of Pt@Fe₃O₄ post magnetic separation from human serum and human saliva. The recovered amount of Pt@Fe₃O₄ was quantified by ICP-MS based on the concentration of Pt. Data shown as mean ± S.D., n = 3 independent magnetic separation experiments of Pt@Fe₃O₄ from human serum and human saliva, respectively, demonstrating the reproducibility of the magnetic separation protocol.* ***(J)*** *Time-resolved evolution of the absorbance at 650 nm caused by the oxidation of TMB in the presence of H_2_O_2_ catalyzed by Pt@Fe₃O₄ post magnetic separation from PBST (DPBS +0.05 v% Tween-20), running buffer (FBS+1 wt% PVP, 10 kDa), human serum and human saliva. DPBS was used as the control condition. Data shown as mean ± S.D., n = 3 independent magnetic separation procedures with a nanoparticle concentration of 1.5 pM.*

*
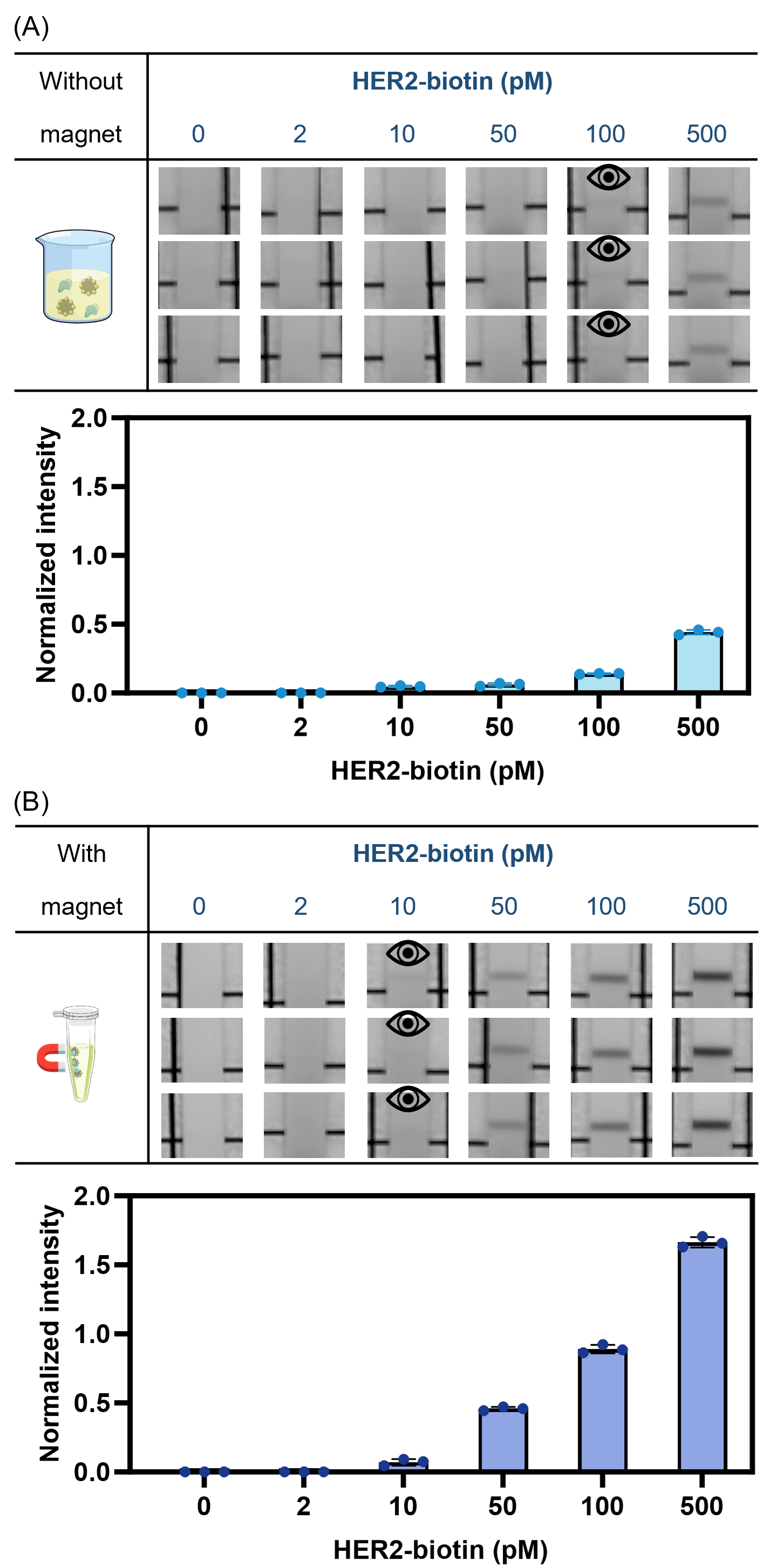
*

***Figure S3*** *Detection of HER2-biotin with Pt@Fe₃O₄@Ab in running buffer without (A) or with (B) magnetic separation and 20-fold volumetric concentration of the sample. Data shown as mean ± S.D., n = 3 independent experiments (magnetic separation procedures) with different batches of nanoparticles. For each magnetic separation procedure, the particles were separated from 1400 μL of running buffer and resuspended in 70 μL of running buffer. The concentrations shown in the figure represent the initial values, i.e., before magnetic separation and concentration. The eye icons represent the visual LOD in these experiments.*

*
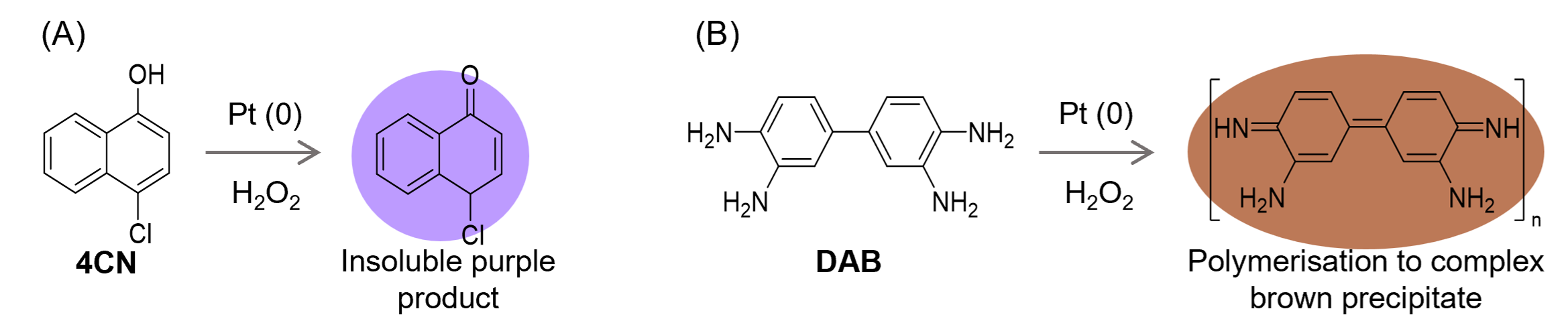
*

***Figure S4 (A)*** *Schematic representation of the oxidation reaction of 4-Chloro-1-naphthol (4CN) catalyzed by Pt(0) in the presence of H_2_O_2_.* ***(B)*** *Schematic representation of the oxidation reaction of 3,3´-Diaminobenzidine (DAB) catalyzed by Pt(0) in the presence of H_2_O_2_.*


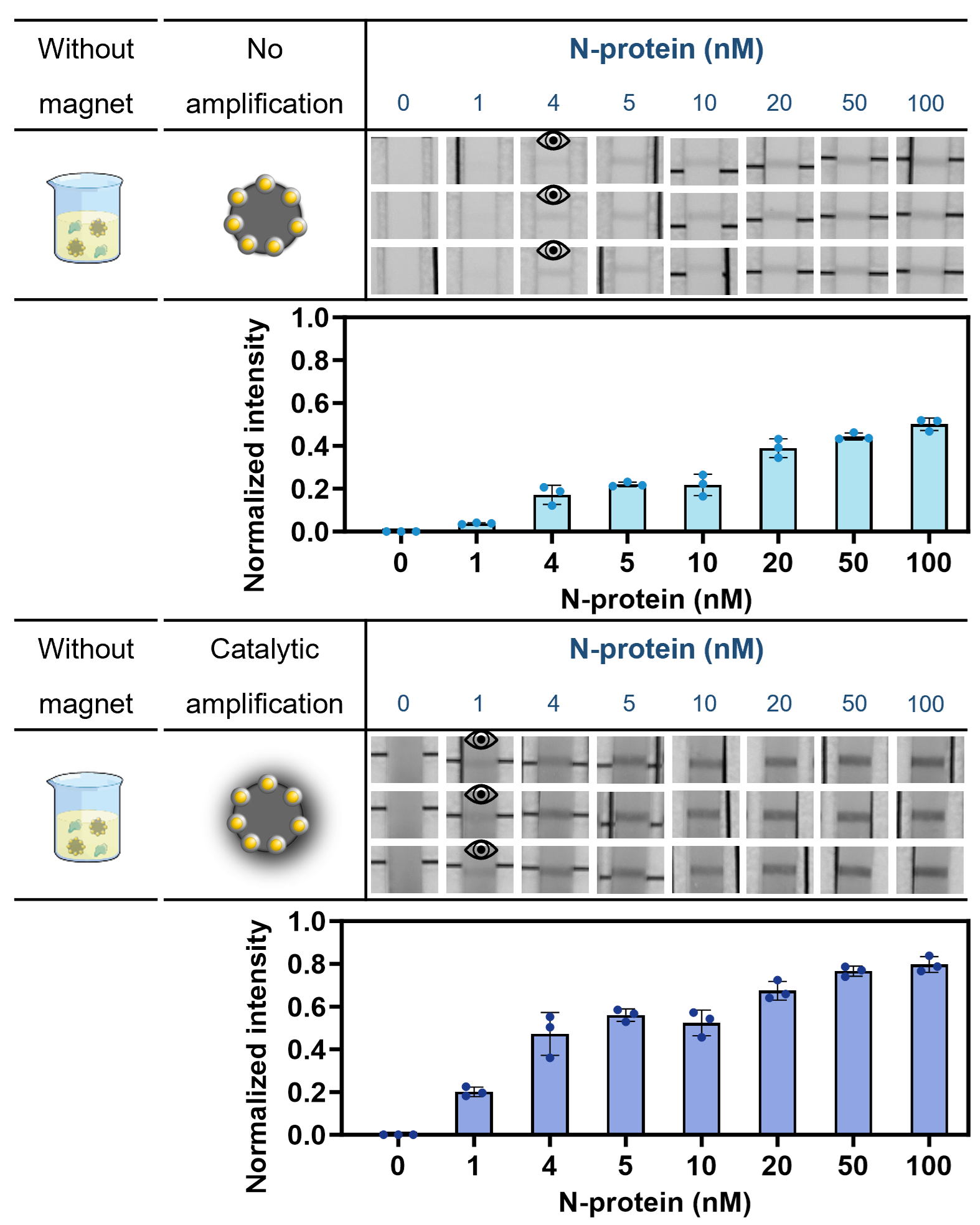


***Figure S5*** *Detection of SARS-CoV-2 N-protein* *with Pt@Fe₃O₄@Ab in running buffer* *without magnetic separation. Data shown as mean ± S.D., n = 3 independent experiments with different batches of nanoparticles. The eye icons represent the visual LOD in these experiments.*


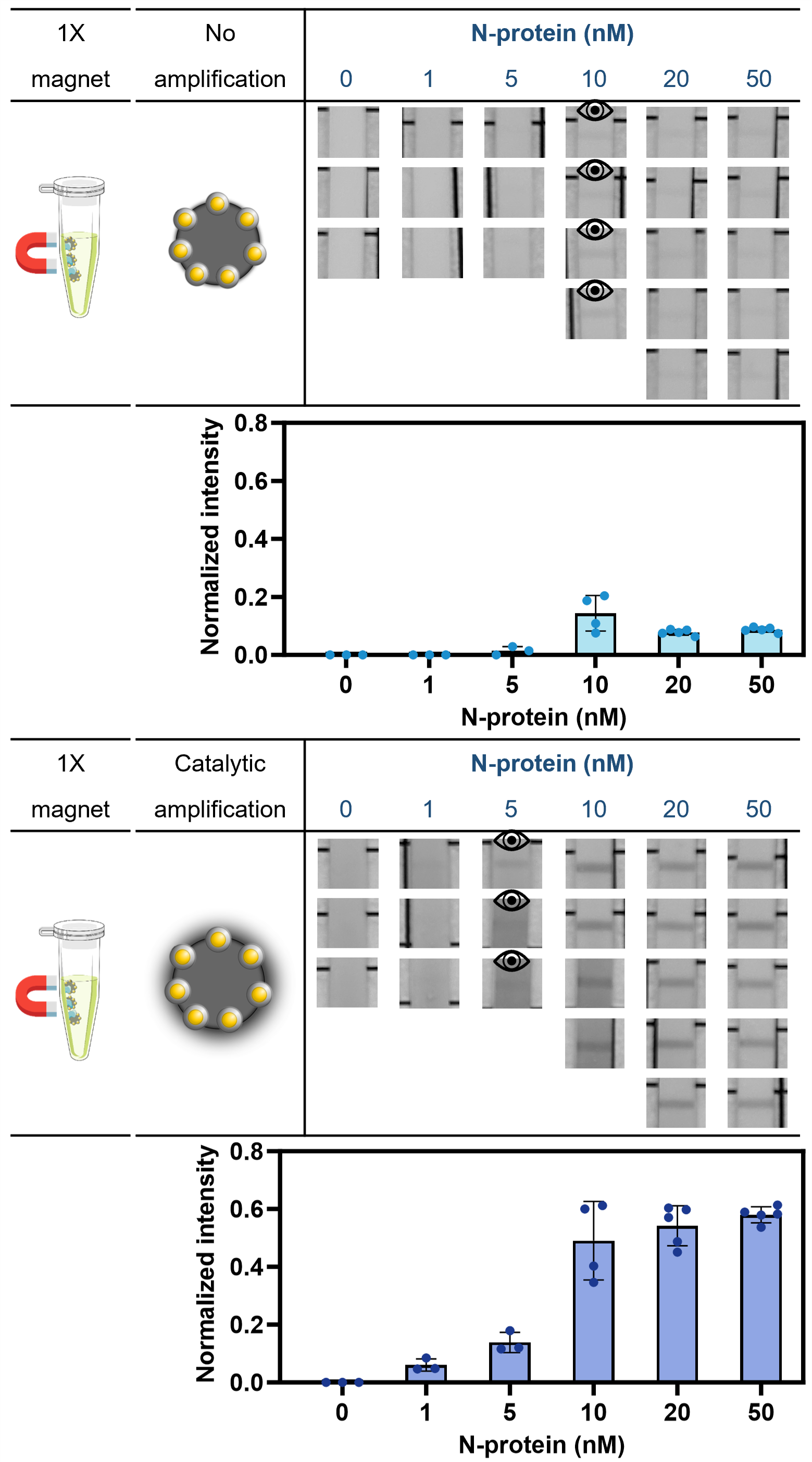


***Figure S6*** *Detection of SARS-CoV-2 N-protein* *with Pt@Fe₃O₄@Ab in running buffer* *with magnetic separation* *but no subsequent volumetric concentration (1×). The particles were separated from 200 μL of running buffer and resuspended in the same amount of runnin buffer. Data shown as mean ± S.D., n ≥ 3 independent magnetic separation procedures with different batches of nanoparticles. The eye icons represent the visual LOD in these experiments.*


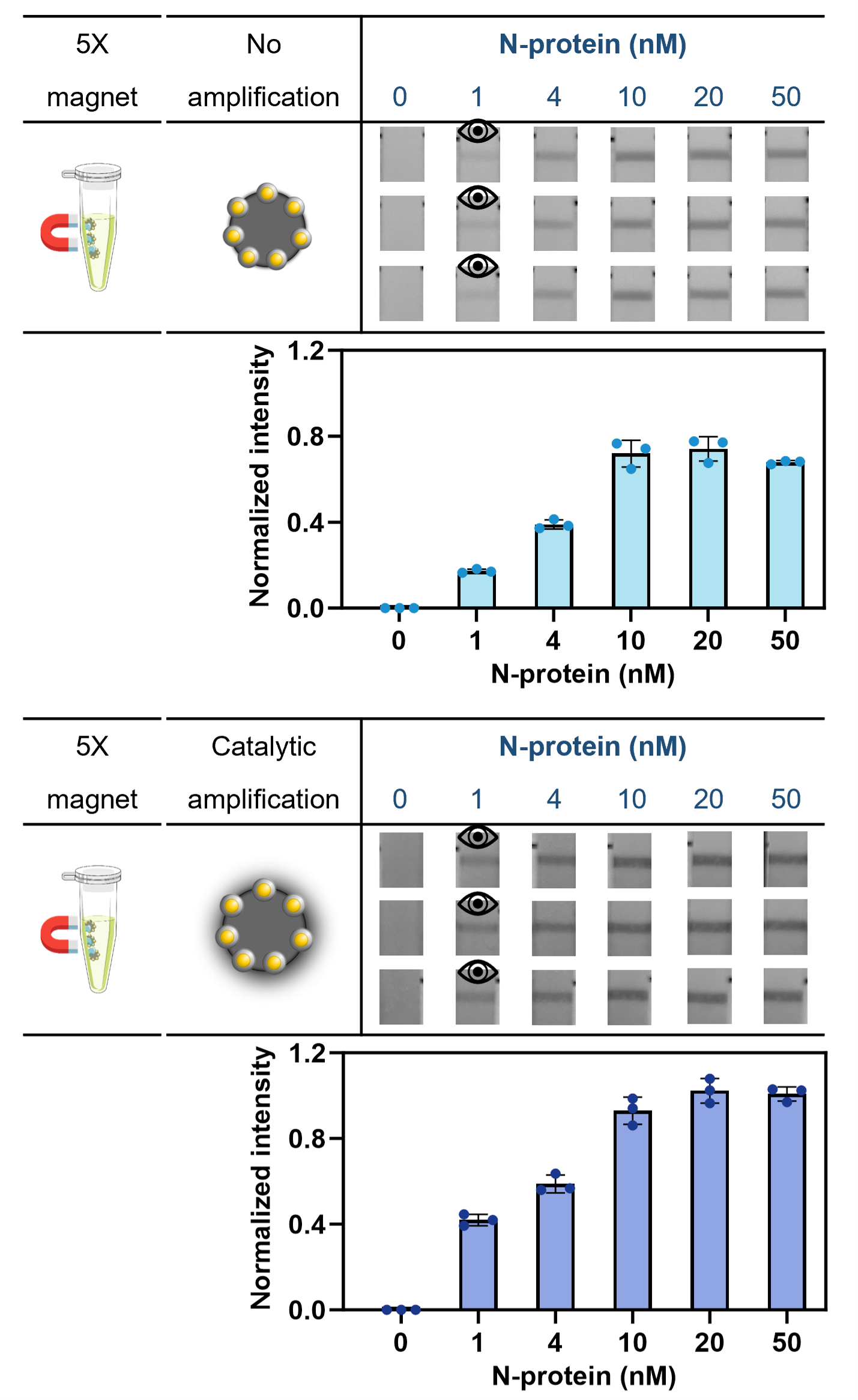


***Figure S7*** *Detection of SARS-CoV-2 N-protein* *with Pt@Fe₃O₄@Ab in running buffer* *with magnetic separation and 5-fold volumetric concentration of the sample (5×). The particles were separated from 350 μL of running buffer and resuspended in 70 μL of running buffer. Data shown as mean ± S.D., n = 3 independent magnetic separation procedures with different batches of nanoparticles. The concentrations shown in the figure represent the initial values, i.e., before magnetic separation and concentration. The eye icons represent the visual LOD in these experiments.*


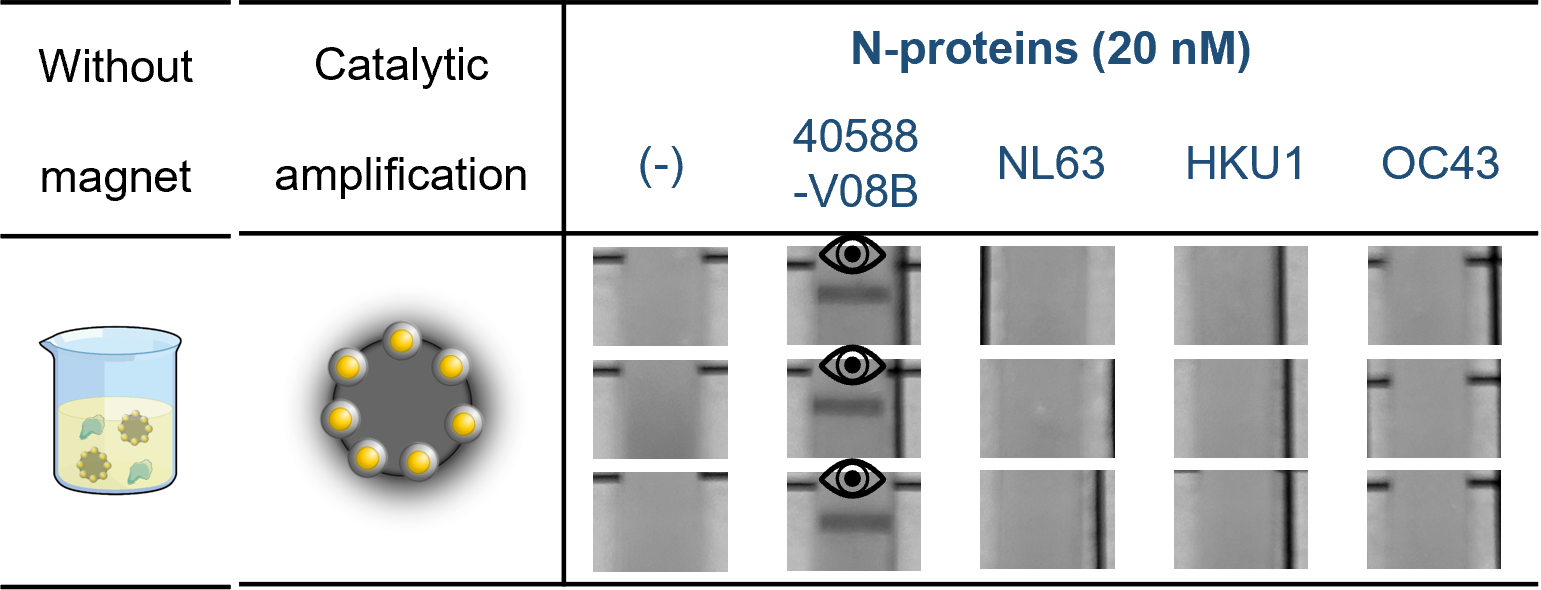


***Figure S8*** *Detection of 20 nM 40588-V08B, NL63, HKU1, and OC43 N-protein* *with Pt@Fe₃O₄@Ab in running buffer* *without magnetic separation and with catalytic amplification. Data from n = 3 independent experiments with different batches of nanoparticles. The eye icons represent the results of positive SARS-CoV-2 N-protein (40588-V08B) applied in this study.*


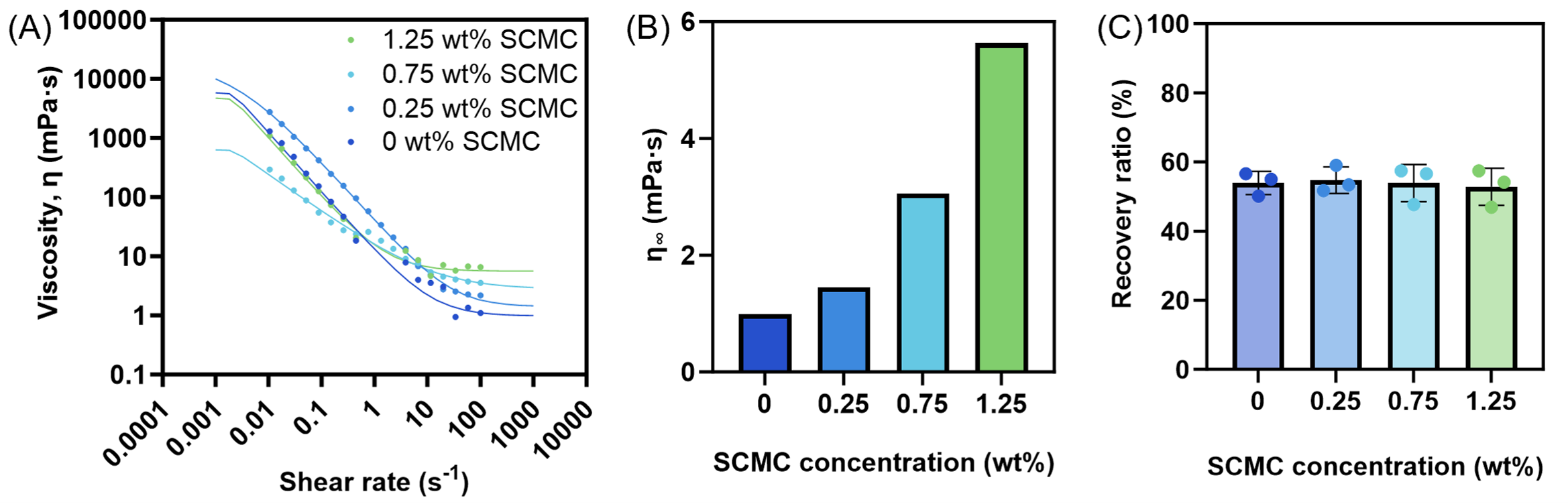


***Figure S9 (A)*** *Viscosity η versus shear rate of artificial saliva containing different concentrations of SCMC (wt%).* ***(B)*** *η_∞_ of artificial saliva containing different concentrations of SCMC (wt%). η_∞_ is the viscosity at 1000 s^-1^ shear rate obtained from regressed viscosity data using the Carreau-Yasuda model.* ***(C)*** *Recovery ratio of Pt@Fe₃O₄ post magnetic separation from artificial saliva with different viscosities. The recovered amount of Pt@Fe₃O₄ was quantified by the UV absorbance of the sample solutions at the wavelength of 500 nm by Nanodrop 2000 (Thermo Fisher Scientific, USA). Data shown as mean ± S.D., n = 3 independent magnetic separation experiments of Pt@Fe₃O₄ from different artificial saliva samples, demonstrating that the recovery ratio and reproducibility of the magnetic separation were not affected by the viscosity of the matrices (Welch’s ANOVA test, p = 0.9764).*


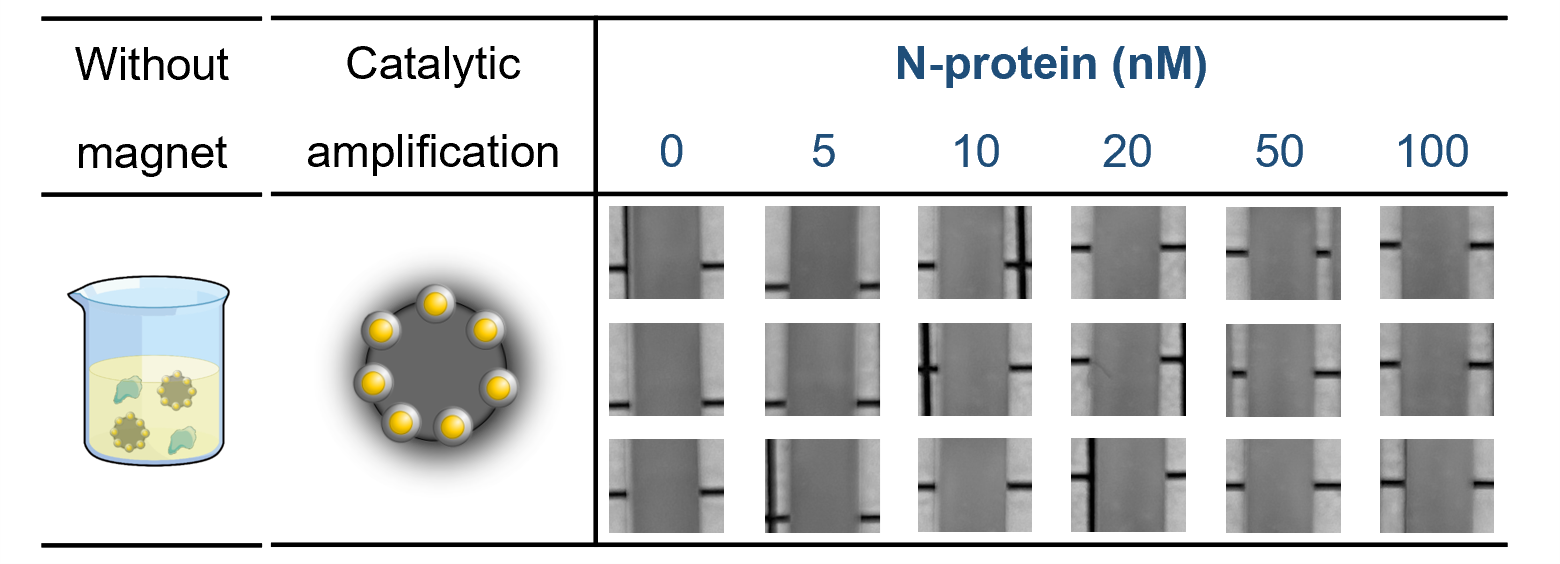


***Figure S10*** *Detection of SARS-CoV-2 N-protein* *with Pt@Fe₃O₄@Ab in human saliva* *without magnetic separation and with catalytic amplification. Data from n = 3 independent experiments with different batches of nanoparticles.*


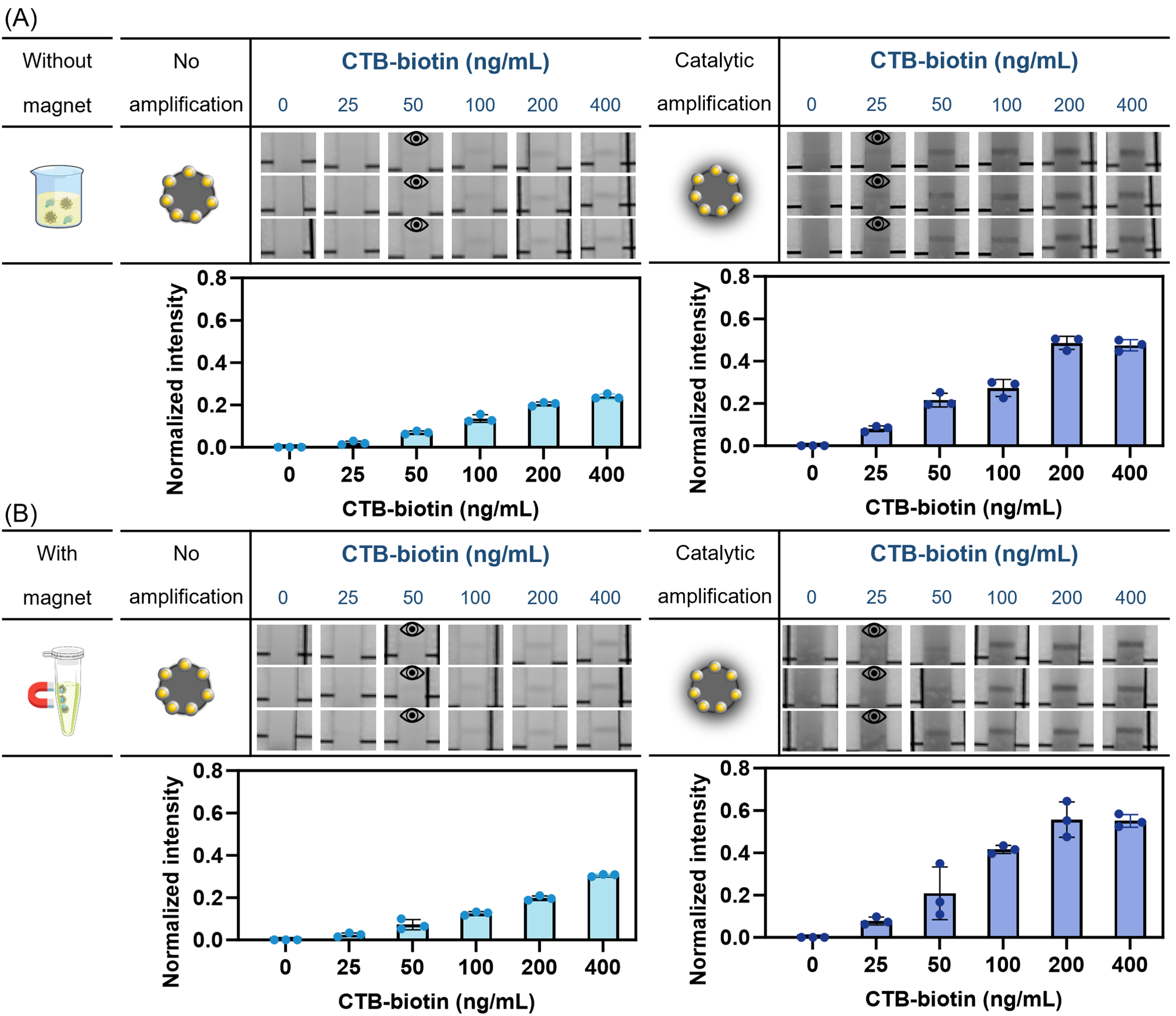


***Figure S11* *(A)*** *Detection of CTB-biotin* *with Pt@Fe_3_O_4_@Ab in running buffer* *without magnetic separation. Data shown as mean ± S.D., n = 3 independent experiments with different batches of nanoparticles.* ***(B)*** *Detection of CTB-biotin* *with Pt@Fe_3_O_4_@Ab in running buffer* *with magnetic separation but no subsequent volumetric concentration. Data shown as mean ± S.D., n = 3 independent magnetic separation procedures with different batches of nanoparticles. For each magnetic separation procedure, the particles were separated from 180 μL of running buffer and resuspended in the same amount of running buffer. The eye icons represent the visual LOD in these experiments.*

**References**

1. Xi, Z.; Wei, K.; Wang, Q.; Kim, M. J.; Sun, S.; Fung, V.; Xia, X., Nickel–Platinum Nanoparticles as Peroxidase Mimics with a Record High Catalytic Efficiency. *J. Am. Chem. Soc.* **2021,** *143* (7), 2660-2664.

2. Jiang, B.; Duan, D.; Gao, L.; Zhou, M.; Fan, K.; Tang, Y.; Xi, J.; Bi, Y.; Tong, Z.; Gao, G. F.; Xie, N.; Tang, A.; Nie, G.; Liang, M.; Yan, X., Standardized assays for determining the catalytic activity and kinetics of peroxidase-like nanozymes. *Nat. Protoc.* **2018,** *13* (7), 1506-1520.

3. Chen, M.; Fan, H.; Li, W.; Ruan, J.; Yang, Y.; Mao, C.; Li, R.; Liu, G. L.; Hu, W., Nanoplasmonic Affinity Analysis System for Molecular Screening Based on Bright-Field Imaging. *Adv. Funct. Mater.* **2024,** *34* (30), 2314481.
